# Unexpectedly minor nitrous oxide emissions from fluvial networks draining permafrost catchments of the East Qinghai-Tibet Plateau

**DOI:** 10.1038/s41467-022-28651-8

**Published:** 2022-02-17

**Authors:** Liwei Zhang, Sibo Zhang, Xinghui Xia, Tom J. Battin, Shaoda Liu, Qingrui Wang, Ran Liu, Zhifeng Yang, Jinren Ni, Emily H. Stanley

**Affiliations:** 1 Key Laboratory of Water and Sediment Sciences of Ministry of Education & State Key Laboratory of Water Environment Simulation, School of Environment, Beijing Normal University, Beijing, China; 2 Guangdong Provincial Key Laboratory of Water Quality Improvement and Ecological Restoration for Watersheds, School of Ecology, Environment and Resources, Guangdong University of Technology, Guangzhou, China; 3 Stream Biofilm and Ecosystem Research Laboratory, School of Architecture, Civil and Environmental Engineering, École Polytechnique Fédérale de Lausanne, Lausanne, Switzerland; 4 Department of Mathematics, Beijing Jiaotong University, Beijing, China; 5 Key Laboratory of Water and Sediment Sciences of Ministry of Education, College of Environmental Sciences and Engineering, Peking University, Beijing, China; 6 Center for Limnology, University of Wisconsin-Madison, Madison, WI USA; 7 Present Address: Sino-French Institute for Earth System Science, College of Urban and Environmental Sciences, Peking University, Beijing, China

**Keywords:** Freshwater ecology, Hydrology, Element cycles

## Abstract

Streams and rivers emit substantial amounts of nitrous oxide (N_2_O) and are therefore an essential component of global nitrogen (N) cycle. Permafrost soils store a large reservoir of dormant N that, upon thawing, can enter fluvial networks and partly degrade to N_2_O, yet the role of waterborne release of N_2_O in permafrost regions is unclear. Here we report N_2_O concentrations and fluxes during different seasons between 2016 and 2018 in four watersheds on the East Qinghai-Tibet Plateau. Thawing permafrost soils are known to emit N_2_O at a high rate, but permafrost rivers draining the East Qinghai-Tibet Plateau behave as unexpectedly minor sources of atmospheric N_2_O. Such low N_2_O fluxes are associated with low riverine dissolved inorganic N (DIN) after terrestrial plant uptake, unfavorable conditions for N_2_O generation via denitrification, and low N_2_O yield due to a small ratio of nitrite reductase: nitrous oxide reductase in these rivers. We estimate fluvial N_2_O emissions of 0.432 − 0.463 Gg N_2_O-N yr^−1^ from permafrost landscapes on the entire Qinghai-Tibet Plateau, which is marginal (~0.15%) given their areal contribution to global streams and rivers (0.7%). However, we suggest that these permafrost-affected rivers can shift from minor sources to strong emitters in the warmer future, likely giving rise to the permafrost non-carbon feedback that intensifies warming.

## Abstract (web summary)

Permafrost soils can be substantial sources of nitrous oxide (N_2_O) to the atmosphere, but no data exist on the N_2_O footprints of permafrost rivers. Here, the authors show that alpine permafrost rivers are unexpectedly small sources of atmospheric N_2_O at present.

## Introduction

Nitrous oxide (N_2_O) is a major stratospheric ozone destroyer and the third most important long-lived greenhouse gas (GHG)^1^. Sources of this powerful GHG are poorly constrained in general, and for global streams and rivers in particular^2^. Studies of lotic N_2_O dynamics have focused almost exclusively on human-impacted lowland systems across diverse climate zones, where anthropogenic nitrogen (N) enrichment has been linked to elevated N_2_O fluxes^3^. Unfortunately, data are extremely sparse for more pristine regions, including the rapidly changing permafrost-rich cryospheres at high altitudes and latitudes. As a result, the current estimate of global fluvial emissions of 291.3 ± 58.6 Gg N_2_O-N yr^−1^ has substantial uncertainty^4^.

Massive amounts of organic carbon (OC, ~1014 Pg C) are stored in the top 3 m of Northern Hemisphere permafrost soils^5^. As these deposits thaw, ice-locked carbon is liberated and can be transported to adjacent running waters, where it becomes available for processing and loss to the atmosphere as carbon dioxide (CO_2_) and methane (CH_4_)^6–9^. Consequently, fluvial emissions of these gases in some cryosphere regions are now occurring at elevated and increasing rates^10,11^. However, the magnitude of N_2_O emissions from streams and rivers in regions where permafrost thaw is afoot is unknown despite the fact that Northern Hemisphere permafrost soils contain 67 Pg N to a depth of 3 m (excluding N pools in the active layer), and are evident or even substantial sources of N_2_O^12^. Studies of Alaskan streams have emphasized sustained delivery of N from thawing permafrost soils resulting in elevated inorganic N^13,14^ and often-supersaturated N_2_O concentrations in these receiving waters^13^. However, in the absence of direct measurements of N_2_O emissions from permafrost-affected streams and rivers, it is not clear if these results are representative and translate to overall elevated N_2_O emissions.

**Fig. 1:**
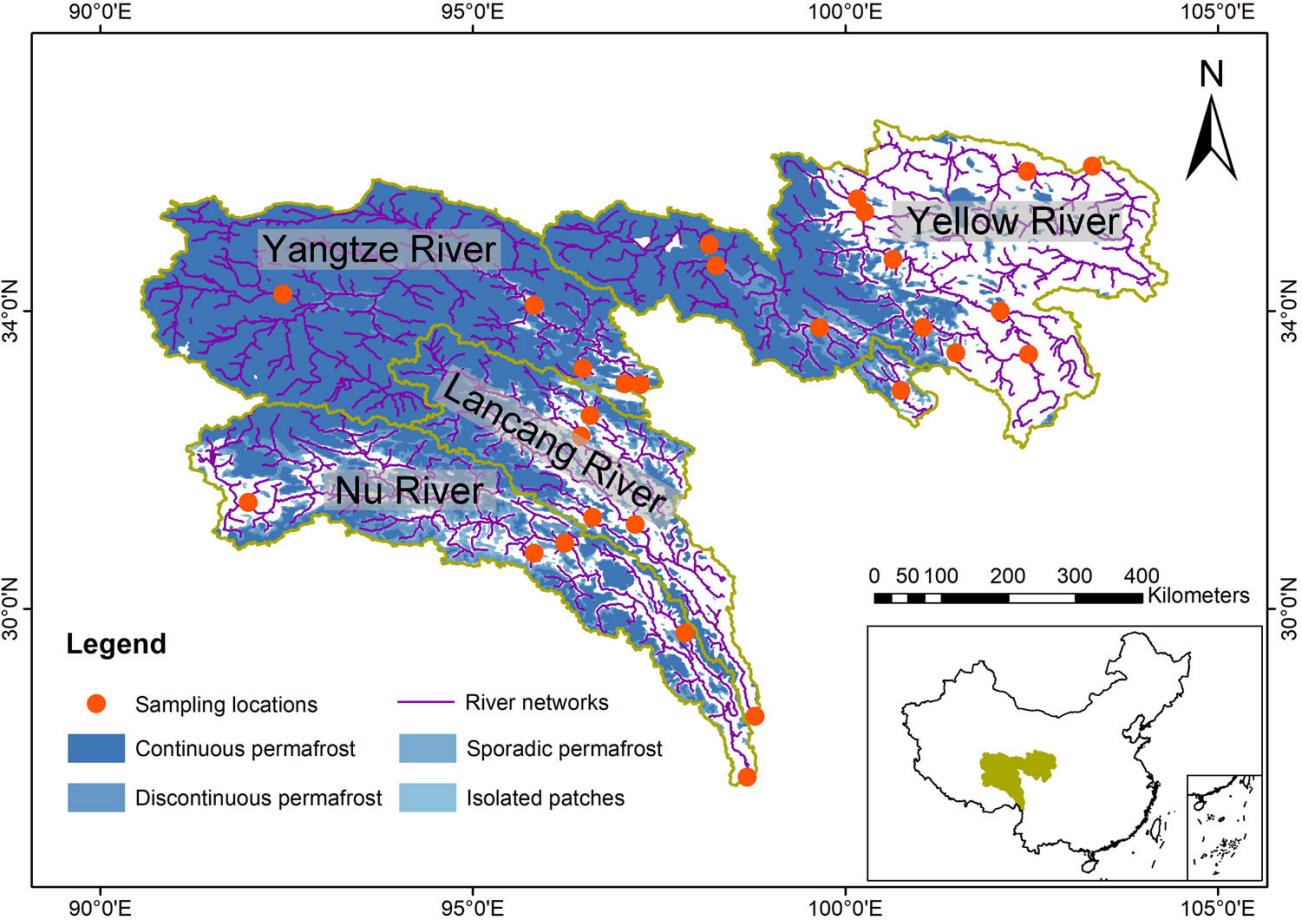
Map of the study sites in four headwater catchments on the East Qinghai-Tibet Plateau (EQTP), China. Sampling sites within the four catchments. The blue shading represents permafrost extent on the EQTP (Data for the permafrost extent courtesy of ref. ^57^). The inset highlights the study area on a map of China.

To address this knowledge gap, we provide a cross-regional and seasonal direct measurement of fluvial N_2_O concentrations and fluxes, generated in four headwater catchments that vary in altitude from 1650 to 4600 m and cover an area of ca. 73.6 × 10^4^ km^2^ on the East Qinghai-Tibet Plateau (EQTP; Fig. 1; Supplementary Fig. 1, Table 1 and 2), and identify likely mechanisms shaping N_2_O dynamics in these rivers. Areal CH_4_ emissions from streams and rivers in this region are strongly affected by permafrost thaw, and are among the highest reported rates globally^11^, suggesting that a similar effect may be underway for N_2_O in these rivers as well. This region is the largest cryosphere outside the Arctic and Antarctic^15^, with vast Pleistocene-aged permafrost^16^ that contains an estimated 1.8 Pg N in the upper 3 m of soil^17^. As the ‘Water Tower of Asia’, meltwater from the Qinghai-Tibet Plateau (QTP) feeds ten great Asian rivers and ubiquitous ponds, lakes, and wetlands^18^. Such alpine streams and rivers are tightly connected to permafrost soils^11^ and have high turbulence-induced gas exchange^19^, and these features potentially promote their capacity to process terrestrial N and sustain high N_2_O emissions. Further, many parts of the QTP have experienced significant human population growth over the last six decades accompanied by similar rapid increases in livestock^20^, again adding to the potential for enhanced riverine N_2_O emissions. However, as we show below, these alpine permafrost-affected streams and rivers of the EQTP are unexpectedly minor atmospheric N_2_O sources. Yet there are signs that developing climate warming and enhanced anthropogenic influences may lead to a substantial increase in fluvial N_2_O emissions from high altitudes and latitudes in the coming decades. This study offers a guide to include permafrost streams and rivers in current and future N_2_O inventories, given their potentially important implications for N_2_O budget.

## Results and discussion

### Variability of N_2_O concentrations and fluxes

All sampled streams and rivers were supersaturated on all dates (117.9–242.5%, *n* = 342 samples from 114 site visits) in N_2_O with respect to the atmosphere. Dissolved N_2_O concentrations fluctuated between 10.2 and 18.9 nmol L^−1^ with an average of 12.4 ± 1.7 nmol L^−1^, which is one-third of the global average^3^ (37.5 nmol L^−1^; Supplementary Table 3). Significantly higher N_2_O concentrations were observed in spring (*P* < 0.001), followed by fall and summer (Supplementary Fig. 2a). Despite differences in catchment attributes including permafrost fraction and population densities (Supplementary Table 2), N_2_O concentrations were not significantly different among the four river systems (Supplementary Fig. 2a).

Diffusive N_2_O fluxes from EQTP rivers were predominantly positive (to the atmosphere), ranging from −14.0 to 40.6 µmol m^−2^ d^−1^ with an average of 9.4 ± 6.2 µmol m^−2^ d^−1^ (*n* = 436 samples from 114 site visits). This mean flux is an order of magnitude lower than the global average^3^ (94.3 µmol m^−2^ d^−1^; Supplementary Table 3). Diffusive N_2_O fluxes were similar in summer and fall, and significantly higher than those in spring (*P* < 0.05; Supplementary Fig. 2b). The asynchronous seasonal patterns between concentrations and fluxes are likely caused by water temperature and precipitation (Supplementary Discussion 1). As with concentration, no significant differences were found for N_2_O diffusion among the four rivers (Supplementary Fig. 2b).

N_2_O ebullition has seldom been documented in lotic ecosystems, although it can coincide with CH_4_ bubble release^21^. Because of the presence of large organic reserves in surrounding permafrost, shallow water depths, and their exposure to low barometric pressure, streams and rivers on the EQTP are notable hotspots of CH_4_ ebullition^11^. Thus, we had hypothesized that N_2_O would also be entrained during the widespread processes of CH_4_ bubble release. The mean N_2_O ebullition rate from EQTP rivers was 0.74 ± 2.47 µmol m^−2^ d^−1^, and accounted for 4.1 ± 11.9% of total N_2_O fluxes (diffusion + ebullition) across all sites. Despite this potential for high ebullition rates, accounting for this flux had a little overall effect, as the average total N_2_O flux (10.2 ± 7.1 µmol m^−2^ d^−1^) was still nine-fold lower than the global mean diffusive N_2_O.

### Terrestrial processes modulating N_2_O dynamics

**Fig. 2:**
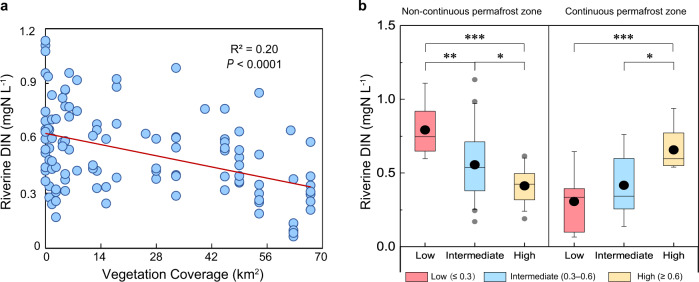
Effect of vegetation on riverine dissolved inorganic nitrogen (DIN). **a** Correlation between vegetation coverage (see Methods) and riverine DIN for all sites across all permafrost categories. The red line represents the fit of a linear regression through the observed data. **b** Riverine DIN for each specific normalized difference vegetation index (NDVI) interval (≤0.3; 0.3–0.6; ≥0.6) in continuous and non-continuous permafrost zones across different seasons (one-way ANOVA with Tukey’s post-hoc test: *P  <  0.05; **P  <  0.01; ***P  <  0.001). Boxes represent the 25^th^ and 75^th^ percentiles, and error bars show the 95^th^ percentiles. Black circles and horizontal lines indicate the arithmetic means and medians, respectively. Gray circles are outliers.

The availability of inorganic N is often the primary determinant of rates of N_2_O production and emission in both permafrost-affected soils^12^ and fluvial networks^2^. In the QTP, dissolved N released from both thawing permafrost soils and animal manure (Supplementary Discussion 2) is biologically available for plant uptake^22–24^ or instead may be exported to river channels. Because plant growth is N-limited in this region^24^, we predicted that an increase in vegetation cover would result in greater plant uptake of terrestrial N, and consequently lower inputs to, and concentrations of N in streams and rivers, up to a point. Indeed, riverine dissolved inorganic N (DIN) concentrations decreased with increasing vegetation cover for all sites (Fig. 2a). However, mechanisms causing the observed decline in riverine DIN cannot be elucidated from vegetation coverage alone, as productivity and greenness per unit plant cover decline at higher elevations^25^. Thus, we also examined normalized difference vegetation index (NDVI, a measure of productivity and greenness) to indicate plant N uptake. Sites with high NDVI had low riverine DIN in non-continuous (namely discontinuous, sporadic, and isolated) permafrost zones (Fig. 2b), in line with the hypothesis of greater plant influence on riverine N availability when and where vegetation productivity and greenness were higher. In contrast, in areas with continuous permafrost, sites with high NDVI had high riverine DIN (Fig. 2b), suggesting that terrestrial N was sufficient to support plant growth and associated productivity/greening regardless of season, and indeed likely exceeded the N demand of plant community^26^. If so, then the surplus DIN can be exported to river corridors. Terrestrial N_2_O can also be transported along with DIN to surrounding watercourses, and higher N_2_O concentrations in some sites draining permafrost areas could be supported by transport of terrestrial N_2_O and/or greater in situ N_2_O production supported by more DIN inputs. Even so, EQTP streams and rivers collectively received reduced terrestrial N after plant uptake. DIN concentrations (0.54 ± 0.30 mgN L^−1^) in EQTP waterways were at the lower end of the range reported for global streams and rivers^3^ (0.002–21.2 mgN L^−1^) and constrained within a relatively narrow range. The low N_2_O concentrations and fluxes are consistent with the low N availability in these rivers.

### Biogeochemical processes regulating N_2_O dynamics

**Fig. 3:**
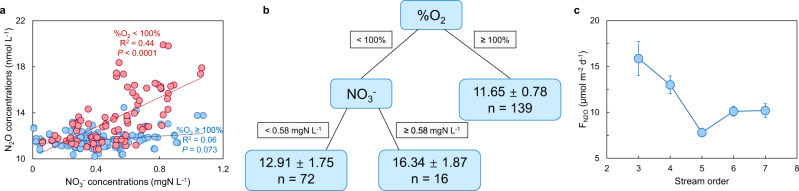
N_2_O as functions of environmental variables. **a** N_2_O concentrations as functions of NO_3_^−^ concentrations for samples with supersaturated O_2_ (blue symbols) and undersaturated O_2_ (red symbols). **b** Regression tree describing predictors of N_2_O concentrations in EQTP rivers. Parameters entering the model were %O_2_ and NO_3_^−^. Values at the ends of each terminal node indicate the N_2_O concentrations (nmol L^−1^ ± 1 SD) and number of observations (n). Cross-validated relative error was 1.70 ± 0.02 and R^2^ was 0.56. **c** ${{{{{{\rm{F}}}}}}}_{{{{{{{\rm{N}}}}}}}_{2}{{{{{\rm{O}}}}}}}$ in relation to stream order across EQTP rivers. All error bars represent ± 1 SE.

Riverine N_2_O concentrations could not be effectively predicted by simple linear regressions with environmental variables [R^2^ ≤ 0.1 in most cases, including dissolved oxygen (DO, *P* > 0.05, R^2^ = 0.004) and NH_4_^+^ concentrations (*P* < 0.001, R^2^ = 0.1); Supplementary Table 4]. However, we found a strong positive relationship between NO_3_^−^ and N_2_O concentrations when DO saturation (%O_2_) was undersaturated (<100%) in the water column (Fig. 3a). This result was validated by a regression tree analysis that identified %O_2_ as the primary control on N_2_O concentration, and that higher N_2_O concentrations occurred when %O_2_ < 100% and NO_3_^−^ ≥ 0.58 mgN L^−1^ (Fig. 3b). The tree had a greater explanatory power (R^2^ = 0.56) than the simple linear regression of NO_3_^−^ and N_2_O concentrations (R^2^ = 0.23; Supplementary Table 4). N_2_O concentrations were uniformly low and weakly related to NO_3_^−^ when %O_2_ ≥ 100% (Fig. 3a), suggesting that N_2_O present at these sites was derived from rare surface sediment patches in the channel that maintain hypoxic-anoxic conditions despite abundant O_2_ in the water column or from external sources including inputs of dissolved N_2_O from permafrost soils, intermediate runoff (Supplementary Fig. 3), and upwelling groundwater^27^ to maintain such low to modest but supersaturated N_2_O concentrations. Regardless of the specific source, this result means that the widespread occurrence of well-oxygenated overlying waters of EQTP rivers (139 of 227 samples) limits the extent of hypoxic-anoxic regimes needed for N_2_O generation via denitrification.

The emission factor EF_5-r_ (EF_5-r_ = N_2_O-N/NO_3_^−^-N) is a surrogate for the conversion of riverine NO_3_^-^ to dissolved N_2_O^28^. Mean EF_5-r_ for EQTP rivers (0.17%) was lower than both the global average (0.22%; Supplementary Table 3) and Intergovernmental Panel on Climate Change (IPCC) default value (0.26%) and is indicative of a small portion of riverine NO_3_^-^ being converted to dissolved N_2_O in EQTP rivers. According to our regression tree analysis, the discrepancies between EF_5-r_ for EQTP rivers and IPCC estimate corroborate past studies that a simple linear NO_3_^−^ model does not adequately predict actual N_2_O^29,30^, because N_2_O concentrations will not necessarily increase with NO_3_^-^ loads in oxic environments. We therefore recommend that the IPCC methodology should be revised to consider nonlinear relationships or interactions among multiple environmental variables.

### Microbial processes underlying N_2_O dynamics

**Fig. 4:**
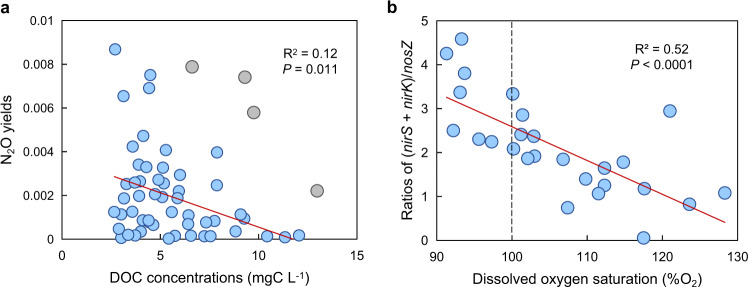
Effect of environmental variables on N_2_O yields and ratios of nitrite reductase: nitrous oxide reductase [(*nirS* + *nirK*)/*nosZ*]. **a** Correlation between dissolved organic carbon (DOC) concentrations and N_2_O yields. Grey points denote samples affected by reservoirs, and were excluded from the correlation analysis. **b** Correlation between dissolved oxygen saturation (%O_2_) and ratios of (*nirS* + *nirK*)/*nosZ*. The red lines represent the fit of a linear regression through the observed data and the vertical dashed line denotes the boundary between super- and sub-saturated dissolved oxygen.

N_2_O yield [ΔN_2_O/(ΔN_2_O + ΔN_2_) × 100%] is a useful metric of relative N_2_O generation^27^, and in EQTP rivers, the N_2_O yield (0.003–0.87%, average 0.23%) was 11 times lower than has been reported for lotic settings (0.01–53.8%, average 2.47%; Supplementary Table 5). This small percentage indicates that N processing in EQTP streams and rivers predominantly generates dinitrogen (N_2_) instead of N_2_O. Laboratory determination of benthic N_2_O production rates confirmed the consistent conversion of N_2_O to N_2_, as these rates were negative (N_2_O was consumed) for two-thirds of the sites (Supplementary Table 6). Low N_2_O yields have been associated with the availability of ample OC to support the complete reduction of NO_3_^−^ to N_2_^2^, and in addition to supplying N, thawing permafrost is also a source of biolabile OC to EQTP streams and rivers^11^ (Fig. 4a).

Examination of relative gene abundances involved in N_2_O production and consumption provides further insights regarding the reason for the low N_2_O yield in EQTP rivers. The key enzymes for N_2_O production are two types of nitrite reductase (*nirS* and *nirK*)^31^, and N_2_O consumption is mediated by clade I and II nitrous oxide reductase (*nosZ*) which catalyzes N_2_O reduction to N_2_^32^. A high ratio of (*nirS* + *nirK*)/*nosZ* indicates an amplified capacity for N_2_O production relative to its loss, leading to high N_2_O concentrations in the water column. This ratio for EQTP riverbed sediments (average 1.96) was far below values reported from other lotic settings worldwide that vary from 2.16 to 3.24 × 10^6^ (average 19.8; Supplementary Table 7), providing compelling evidence for a molecular basis for the low N_2_O yield in EQTP rivers. Unexpectedly, we also found a negative correlation between the (*nirS* + *nirK*)/*nosZ* ratio and %O_2_ (Fig. 4b; Supplementary Discussion 3), illustrating that the microbial community increasingly favors the reduction of N_2_O to N_2_ as DO saturation increased^33^.

### Physicochemical processes governing N_2_O dynamics

To understand potential controls on N_2_O fluxes (${{{{{{\rm{F}}}}}}}_{{{{{{{\rm{N}}}}}}}_{2}{{{{{\rm{O}}}}}}}$), we used stepwise regression to assess the relationships between ${{{{{{\rm{F}}}}}}}_{{{{{{{\rm{N}}}}}}}_{2}{{{{{\rm{O}}}}}}}$ and multiple environmental variables known to influence ${{{{{{\rm{F}}}}}}}_{{{{{{{\rm{N}}}}}}}_{2}{{{{{\rm{O}}}}}}}$. The analysis showed that %O_2_, pH, water temperature, total phosphorus, and NO_3_^−^ all had significant but weak relationships with ${{{{{{\rm{F}}}}}}}_{{{{{{{\rm{N}}}}}}}_{2}{{{{{\rm{O}}}}}}}$ (*P* < 0.001, R^2^ = 0.14; Supplementary Table 8).

Local hydrogeomorphology is, at least qualitatively, a reliable predictor of ${{{{{{\rm{F}}}}}}}_{{{{{{{\rm{N}}}}}}}_{2}{{{{{\rm{O}}}}}}}$ downstream trends^2^. Along the longitudinal continuum, ${{{{{{\rm{F}}}}}}}_{{{{{{{\rm{N}}}}}}}_{2}{{{{{\rm{O}}}}}}}$ was highest in 3^rd^-order (headwater) streams, declined in 4^th^- and 5^th^-order (medium-sized) rivers, and was slightly elevated in 6^th^- and 7^th^-order (large) rivers (Fig. 3c). The decline in flux from 3^rd^- to 5^th^-order streams may reflect the reduced perimeter-to-surface-area ratio (ratio of wetted perimeter to cross-sectional area) and hyporheic exchange rates (exchange rates of dissolved substances between surface water and groundwater beneath and alongside the river channel) with increasing stream order^34–36^, while the increase in 6^th^- and 7^th^-order sites might be due to increasing riverine DIN concentrations^2^ (Supplementary Fig. 4). Furthermore, increased suspended sediment loads can enhance N_2_O generation in larger turbid channels, as suspended particles provide micro-niches that support N transformations^37^, and thus facilitate N_2_O production in the water column^2,36^. Suspended sediment concentration increased with stream order for these rivers^11^, lending support to the hypothesis that this mechanism contributes to higher fluxes observed in 6^th^- and 7^th^-order channels.

### Regional and global implications

Based on our flux measurements, we estimated that EQTP 3^rd^- to 7^th^-order streams and rivers emitted 0.206 Gg N_2_O-N yr^−1^ (5–95^th^ percentiles: 0.129–0.291 Gg N_2_O-N yr^−1^, 2603 km^2^ of river channel area). Our upscaling did not include 1^st^- and 2^nd^-order streams, which can contribute disproportionately high areal fluxes to overall fluvial N_2_O emissions^36,38^. Low-order streams are always well connected to continuous permafrost and hence should receive high N inputs while having reduced N_2_O solubility owing to high altitudes, and these conditions are expected to lead to high N_2_O fluxes. Based on this logic, we estimated a total emission of 0.275 Gg N_2_O-N yr^−1^ from 1^st^- to 7^th^-order streams and rivers (5–95^th^ percentiles: 0.162–0.400 Gg N_2_O-N yr^−1^, 3049 km^2^) by extrapolating the relationship in Fig. 3c to include 1^st^- and 2^nd^-order streams.

Despite large uncertainties due to a lack of observational data from 1^st^- and 2^nd^-order streams (Supplementary Discussion 4), the upscaling exercise enables us to place our estimates in a broader context of both regional N_2_O budgets and fluvial emissions at the global scale. The percentage of N_2_O in total GHG (CO_2_ + CH_4_ + N_2_O) emissions expressed as CO_2_ equivalents corresponded to 1.0% for EQTP 3^rd^- to 7^th^-order drainages, then dwindled to 0.4% for EQTP 1^st^- to 7^th^-order streams and rivers, falling within the range of pristine rivers (0.2–1.2%)^39,40^. These values contrast those from human-impacted fluvial networks, where N_2_O percentages are generally much higher (2.8–13.9%, average 6.8%; Supplementary Table 9) due to often elevated inputs of fertilizer- or sewage-derived N that boost N_2_O emissions. Expressed as per unit stream/river and basin area, EQTP 1^st^- to 7^th^-order streams and rivers released a total of 0.08 t N_2_O-N km^−2^ yr^−1^ and 0.32 kg N_2_O-N km^−2^ yr^−1^, respectively, to the atmosphere, which are one order of magnitude lower than those from lotic systems worldwide [0.65 (range: 0.08–2.55) t N_2_O-N km^−2^ yr^−1^ and 2.44 (range: 0.55–5.78) kg N_2_O-N km^−2^ yr^−1^, respectively; Supplementary Table 9]. Applying the emission rate per unit stream/river and basin area to the entire QTP 1^st^- to 7^th^-order drainage networks, we obtained a riverine N_2_O emission of 0.432–0.463 Gg N_2_O-N yr^−1^, which is minor (~0.15%) given their areal contribution (0.7%) to global streams and rivers^41^. In addition, these N_2_O estimates are probably overestimated. The emission of N_2_O accumulated underneath the ice during winter was estimated to be 15% of the annual emission^39^. This ice-melt outgassing of winter N_2_O was included in the above annual N_2_O emissions; however, this flux may be very limited in permafrost-affected systems due to minimum N inputs from frozen soils in winter^42^. These alpine permafrost waterways emit large amounts of CH_4_^11^, but fortunately they are currently small contributors of N_2_O delivery to the atmosphere, demonstrating CH_4_ and N_2_O dynamics are uncoupled within these systems.

Although QTP fluxes were small, existing global estimates do not effectively capture this natural fluvial source of N_2_O, nor are the major drivers of N_2_O dynamics well known for these systems. Our study is a step forward in quantifying fluvial N_2_O evasion from a cryospheric biome, and highlights the unique dynamic nature of N_2_O concentrations and fluxes in high-altitude environments. Our finding that oxygen saturation was the first and primary correlate of N_2_O concentrations may also have broader implications for aquatic N_2_O dynamics in other high-altitudinal streams.

### Future fluvial N_2_O emissions under warming climate

**Fig. 5:**
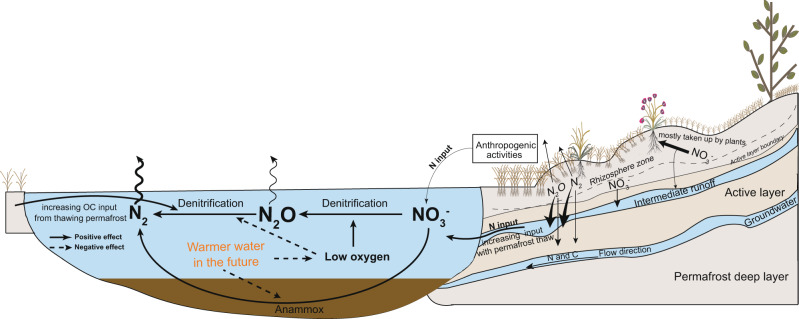
Conceptual model of potential fluvial N and/or N_2_O processes and pathways with deeper thaw in a warmer climate. Future warming and associated permafrost thaw, together with enhanced human perturbations will increase terrestrial N input into surrounding river networks. Warming also raises water temperatures (highlighted in orange), promoting N_2_O production via denitrification with suppressing the reduction of N_2_O to N_2_, eventually resulting in elevated N_2_O emissions.

Temperatures are rising faster in high altitudes and latitudes than in other regions^43^. As warming continues, permafrost thaw is expected to increase, liberating substantial amounts of dissolved N^12^. As this process progresses into deeper soil layers below the rhizosphere, diminished plant uptake^24^ should favor greater export of N to streamflow via deep flow paths^14,44,45^. Meanwhile, warmer water temperatures reduce gas solubility and enhance hypoxia and denitrification at the expense of anammox^46^, directing more N towards denitrification, and concomitant N_2_O production and evasion to the atmosphere (Fig. 5). Moreover, the duration of the ice-free season across the cryosphere is rapidly increasing and will continue to increase^47^, suggesting a potential proxy for riverine N_2_O release. Furthermore, human perturbations may bring an extra N burden to the cryosphere and exacerbate these impacts. Taken together, these processes might render streams and rivers draining permafrost catchments across the globe to become hotspots of N_2_O to the atmosphere in the future, leading to positive non-carbon climate feedback of currently unanticipated magnitude because of an increase in fluvial N_2_O production following the development of climate change and escalation of anthropogenic influence.

The high degree of spatiotemporal variability in riverine N_2_O observed here is likely to exist in other unexplored cryospheres. Further progress in understanding how aquatic ecosystems in these climate-sensitive regions will respond to ongoing global warming would benefit greatly from more N_2_O measurements from glacial and permafrost-affected lotic and lentic systems at high spatial and temporal resolution. Alongside N_2_O measurements, capturing the fate of thawed N in cryospheric aquatic systems are indispensable to stitching together pathways and processes into a holistic framework. It is time to improve our understanding of cryospheric aquatic N_2_O emissions in shaping the global N_2_O budget, and how this contribution might be altered in progressively warming high altitudes and latitudes.

## Methods

### Site description

The ~73.6 × 10^4^ km^2^ study area of the East Qinghai-Tibet Plateau (EQTP) is located between the boundary of the Loess Plateau (Gansu Province, 36° N) and the southern frigid limit (Yunnan Province, 28° N). This region is characterized by a high-relief landscape ranging from 1650 to 7000 m above sea level. The elevational gradient, along with abundant but variably distributed permafrost creates geomorphological, hydrological, vegetative, and climatological heterogeneities in the alpine landscape. The EQTP includes four great Asian rivers—the Yangtze, Yellow, Lancang-Mekong and Nu-Salween Rivers. We sampled a broad range of streams and rivers in these basins that ranged from Strahler order 3 to 7. All sampling sites were visited during the daytime in spring (May–June), summer (July–August), and fall (September–October) between 2016 and 2018. The Yellow River was sampled seven times, the Yangtze River four times, and the Lancang and Nu Rivers were both sampled on three dates. We grouped the sampling sites into four categories that represent different permafrost zones: continuous, discontinuous, sporadic, and isolated (Fig. 1). We then merged discontinuous, sporadic, and isolated permafrost zones together under the non-continuous permafrost group in response to different NDVI trends.

### N_2_O concentrations and fluxes

Triplicate samples for dissolved N_2_O concentrations were collected by completely filling 120 mL glass serum bottles at wrist depth below the water surface at each site. After preserving samples with 0.5 mL saturated ZnCl_2_ solution, the serum bottles were sealed with butyl stoppers, crimped with aluminum caps, and stored at ambient temperature in the dark. Local ambient air samples were also taken and used to back-calculate N_2_O concentration in water in equilibrium with the atmosphere. N_2_O concentrations were determined via the headspace equilibration method^48^ on a gas chromatography equipped with an electron capture detection for N_2_O (Agilent 7890B GC-µECD). The partial pressure of CO_2_ (*p*CO_2_) in surface water was determined following our earlier work^11^. Sampling and analysis of dissolved N_2_ concentrations are described in Supplementary Methods 1.

${{{{{{\rm{F}}}}}}}_{{{{{{{\rm{N}}}}}}}_{2}{{{{{\rm{O}}}}}}}$ was measured simultaneously with dissolved N_2_O concentration collection. Four floating chambers were held in place at each transect, covering depth gradients from the river bank to the mid-channel to capture the spatial heterogeneity. Measurements lasted for 1 h at each site, and 50 mL gas extracted from inside the chambers at 0, 5, 10, 20, 40, and 60 min intervals were injected into air-tight gas sampling bags for analysis in the laboratory by GC-µECD. These chambers were of the same size and shape and streamlined with a flexible plastic foil collar to minimize the effects of chamber-induced turbulence when measuring fluxes^49^ and were covered with aluminum foil to reflect the sunlight and minimize internal heating.

Surface water and sediment samples were collected simultaneously with gas samples for physicochemical and microbial analyses at each site, respectively. Air temperature, air pressure, and wind speed were measured in situ with a portable anemometer (Testo 480). DO, pH, ORP, conductivity, and water temperature were measured in situ with portable field probes (Hach HQ40d). Annual air temperature and precipitation were obtained from the National Meteorological Information Center (http://data.cma.cn/).

### Flux computation

Total ${{{{{{\rm{F}}}}}}}_{{{{{{{\rm{N}}}}}}}_{2}{{{{{\rm{O}}}}}}}$ were calculated according to the equation below:

1 $${F}_{{{{{{\rm{t}}}}}}}=\frac{{n}_{{{\mbox{t}}}}{{\mbox{-}}}{n}_{0}}{A\times t}$$

where, *n*_t_ and *n*_0_ are the number of moles of N_2_O in the chamber at time t and time zero (mol), respectively; *A* is the surface area of water covered by the chamber (m^2^) and *t* is the measurement duration time (min). Diffusive and ebullitive N_2_O fluxes were separated using the Campeau et al. approach^50^. Briefly, we assumed that *F*_t_ for CO_2_ (F_CO2_) is exclusively diffusive (that is, CO_2_ ebullition is negligible). F_CO2_ was computed from the linear regression of *p*CO_2_ against time to eliminate possible bias due to gas accumulation in the chamber headspace that can affect the flux rates. We then used F_CO2_ to calculate *k*_CO2_ by rearranging the equation for Fick’s law of gas diffusion^51^:

2 $${k}_{{{{{{\rm{CO}}}}}}2}={F}_{{{{{{\rm{CO}}}}}}2}/({K}_{{{{{{\rm{H}}}}}}}\Delta p{{CO}}_{2})$$

where *K*_*H*_ is the temperature-adjusted Henry’s constant, and Δ*pCO*_*2*_ is the CO_2_ partial pressure difference between surface water and the atmosphere. Finally, the theoretical diffusive *k* for N_2_O was calculated based upon *k*_CO2_ as follows^52^:

3 $${k}_{{{{{{\rm{N}}}}}}2{{{{{\rm{O}}}}}}}/{k}_{{{{{{\rm{CO}}}}}}2}={({{Sc}}_{{{{{{\rm{N}}}}}}2{{{{{\rm{O}}}}}}}/{{Sc}}_{{{{{{\rm{C}}}}}}{{{{{\rm{O}}}}}}2})}^{-n}$$

where *k*_N2O_ and *k*_CO2_ are gas transfer velocity of N_2_O and CO_2_, respectively; *Sc* is the Schmidt number. *n* = 1/2 is used for rippled or turbulent surface water conditions, or *n* = 2/3 is used for a smooth water surface^53^. We then calculated the theoretical diffusive ${{{{{{\rm{F}}}}}}}_{{{{{{{\rm{N}}}}}}}_{2}{{{{{\rm{O}}}}}}}$ according to

4 $${F}_{{{{{{\rm{d}}}}}}}=k({C}_{{{{{{\rm{water}}}}}}}{{{{{\rm{\hbox{-}}}}}}}{C}_{{{{{{\rm{eq}}}}}}})$$

where *k* is gas transfer velocity (m·d^−1^), *C*_water_ is water gas concentration (mol m^−3^), and *C*_eq_ is gas concentration in water in equilibrium with the local atmosphere corrected for temperature-induced changes in solubility according to the Henry’s law (mol m^−3^). Thus, the difference between the total and diffusive N_2_O fluxes is attributable to ebullition.

### Physicochemical analyses

Duplicate filtered water samples were analyzed for NH_4_^+^-N, NO_2_^−^-N, and NO_3_^−^-N, as well as dissolved organic carbon (DOC) following detailed methods described in ref. ^11^. Total phosphorus (TP) was measured on a UV − Vis spectrophotometer (Agilent 1200) using the standard molybdenum blue method after persulfate digestion. Determination of sedimentary N_2_O production rates is described in Supplementary Methods 2.

### Microbial analyses

A total of 26 sediment samples collected across the four rivers between 2016 and 2018 were prepared in triplicate. Genomic DNA was extracted from approximately 0.5 g fresh homogenized sediment using the FastDNA^®^ SPIN Kit for Soil (MP Biomedicals), following the manufacturer’s instructions. Abundances of dissimilatory nitrite reductase (*nirS* and *nirK*)^31^ and nitrous oxide reductase (*nosZ*_I_ and nos*Z*_II_) genes^32^ in these river sediments were estimated by real-time quantitative PCR. Detailed information is available in Supplementary Methods 3.

### GIS analyses

Vegetation types in the study area are alpine meadow and steppe, and vegetation coverage for each type was extracted using datasets from ref. ^54^. The vegetation coverage for each river reach was determined within a circular area of 5 km in radius centered around each sampling site using the ArcGIS 10.6 buffer analysis tool. Normalized difference vegetation index (NDVI) data were obtained from the China Monthly Vegetation Index Spatial Distribution Data Set (Resource and Environment Science and Data Center, Chinese Academy of Sciences, https://www.resdc.cn/), with a spatial resolution of 1 × 1 km^2^ for the period 2016–2018 corresponding to sampling dates. The NDVI values for each river reach were calculated as the mean value within a circular area of 5 km in radius centered around each sampling site using the ArcGIS 10.6 zonal statistics tool.

Hydrological analysis was performed on the ArcGIS 10.4 platform. Streams and rivers were extracted and Strahler stream order for all stream lines in the four catchments was calculated based on Digital Elevation Model (DEM) data. We then calculated the total surface area for each stream order by multiplying the total length with the average width of this stream order. The total length was derived from the distance tool in the ArcGIS. We calculated the average river width for each stream order based on in situ width measurements at the sampling location and 50 additional locations of the corresponding stream order from Google Earth Maps in each catchment. Average stream width for the 1^st^- and 2^nd^-order streams were obtained from the width ratios found for rivers of high stream orders according to our earlier work^11^.

### Upscaling technique

We upscaled the magnitude of N_2_O emissions (and uncertainty) from EQTP streams and rivers with a Monte Carlo simulation (MATLAB R2018b) that ran 1000 iterations for each sampled 3^rd^- to 7^th^-stream order. Each iteration randomly selected a N_2_O flux measurement and simultaneously selected a surface area based on a normal distribution surrounding the mean and standard deviation for that stream in order to generate an order-specific N_2_O flux per unit of time. This value was then summed across the ice-free season (210 d from April to October) to estimate N_2_O emission for each stream order. For 1^st^- and 2^nd^-order streams, a range of N_2_O fluxes that extrapolated from the relationship in Fig. 3c and the surface area for this order were used to produce total fluxes and constrain the uncertainty. We summed up the final distribution of N_2_O emissions from stream orders 1–7, including the mean value and 95% confidence intervals. Finally, we divided the ice-free season emission by 85% to obtain the annual emission. This is because early spring efflux of winter-derived N_2_O fuels ~15% of the annual emissions^39^.

### Statistical analyses

Pearson correlations and multiple linear stepwise regression analysis were conducted under the Statistical Product and Service Solutions 25.0 software (SPSS) at a significance level of *P* < 0.05. To uncover complex dependencies among predictor variables, we used a regression tree approach to analyze predictors of N_2_O concentrations with MATLAB R2018b. This nonparametric method does not require linear relationships and allows for interaction effects among predictors. All data form a single group at the top of the tree, and the tree is grown by repeatedly splitting the data into two subgroups, with each split based on the explanatory variable which makes the resulting groups as different as possible. All values of predictive variables are assessed each time a dichotomy is made^55^. For each of the two subgroups, the same process was repeated until a tree is formed. Such a tree needed to be pruned to avoid overfitting and improve predictive accuracy. We then applied 10-fold cross-validation and pruned the tree based on the ‘1-SE’ rule. This was a parsimonious approach to find the smallest tree whose cross-validated relative error (CVRE) is within one standard error of the minimum. We report the amount of variance explained as R^2^ to depict the fit of the tree. We also report the CVRE since it is a suitable measure of prediction^56^. Predictive variables available for entry into the model were: %O_2_ and NO_3_^-^. Given the high CVRE here, the model is more suggestive than predictive.

### Reporting summary

Further information on research design is available in the Nature Research Reporting Summary linked to this article.

## Supplementary information

Supplementary Information

Peer Review File

Reporting Summary

## Data Availability

The data used in this study are available at the Environmental Data Initiative (https://portal.edirepository.org/nis/mapbrowse?packageid=edi.695.1), 10.6073/pasta/ba9340800403c450e7d942d450237dc4.

## Code availability

The Monte Carlo model and regression tree analysis code used in this study are available at the Environmental Data Initiative (https://portal.edirepository.org/nis/mapbrowse?packageid=edi.695.1), 10.6073/pasta/ba9340800403c450e7d942d450237dc4

## References

[1] Ravishankara AR, Daniel JS, Portmann RW (2009). Nitrous oxide (N_2_O): the dominant ozone-depleting substance emitted in the 21^st^ century. Science.

[2] Quick AM (2019). Nitrous oxide from streams and rivers: a review of primary biogeochemical pathways and environmental variables. Earth Sci. Rev..

[3] Hu M, Chen D, Dahlgren RA (2016). Modeling nitrous oxide emission from rivers: a global assessment. Glob. Change Biol..

[4] Yao Y (2020). Increased global nitrous oxide emissions from streams and rivers in the Anthropocene. Nat. Clim. Change.

[5] Mishra U (2021). Spatial heterogeneity and environmental predictors of permafrost region soil organic carbon stocks. Sci. Adv..

[6] Striegl, R. G., Dornblaser, M. M., McDonald, C. P., Rover, J. R. & Stets, E. G. Carbon dioxide and methane emissions from the Yukon River system. *Glob. Biogeochem. Cycles*10.1029/2012gb004306 (2012).

[7] Crawford JT, Striegl RG, Wickland KP, Dornblaser MM, Stanley EH (2013). Emissions of carbon dioxide and methane from a headwater stream network of interior Alaska. J. Geophys. Res. Biogeosci..

[8] Zolkos S, Tank SE, Striegl RG, Kokelj SV (2019). Thermokarst effects on carbon dioxide and methane fluxes in streams on the Peel Plateau (NWT, Canada). J. Geophys. Res. Biogeosci..

[9] Dean JF (2020). East Siberian Arctic inland waters emit mostly contemporary carbon. Nat. Commun..

[10] Serikova S (2018). High riverine CO_2_ emissions at the permafrost boundary of Western Siberia. Nat. Geosci..

[11] Zhang L (2020). Significant methane ebullition from alpine permafrost rivers on the East Qinghai-Tibet Plateau. Nat. Geosci..

[12] Voigt C (2020). Nitrous oxide emissions from permafrost-affected soils. Nat. Rev. Earth Environ..

[13] Abbott BW, Jones JB, Godsey SE, Larouche JR, Bowden WB (2015). Patterns and persistence of hydrologic carbon and nutrient export from collapsing upland permafrost. Biogeosciences.

[14] Khosh MS (2017). Seasonality of dissolved nitrogen from spring melt to fall freezeup in Alaskan Arctic tundra and mountain streams. J. Geophys. Res. Biogeosci..

[15] Yang M, Wang X, Pang G, Wan G, Liu Z (2019). The Tibetan Plateau cryosphere: observations and model simulations for current status and recent changes. Earth Sci. Rev..

[16] Jin, H. J., Chang, X. L. & Wang, S. L. Evolution of permafrost on the Qinghai-Xizang (Tibet) Plateau since the end of the late Pleistocene. *J. Geophys. Res. Earth Surface*10.1029/2006jf000521 (2007).

[17] Kou D (2019). Spatially-explicit estimate of soil nitrogen stock and its implication for land model across Tibetan alpine permafrost region. Sci. Total Environ..

[18] Immerzeel WW, Bierkens MFP (2012). Asia’s water balance. Nat. Geosci..

[19] Horgby Å (2019). Unexpected large evasion fluxes of carbon dioxide from turbulent streams draining the world’s mountains. Nat. Commun..

[20] Chen H (2013). The impacts of climate change and human activities on biogeochemical cycles on the Qinghai-Tibetan Plateau. Glob. Change Biol..

[21] Baulch, H. M., Dillon, P. J., Maranger, R. & Schiff, S. L. Diffusive and ebullitive transport of methane and nitrous oxide from streams: are bubble-mediated fluxes important? *J. Geophys. Res. Biogeosci.*10.1029/2011jg001656 (2011).

[22] Keuper F (2012). A frozen feast: thawing permafrost increases plant-available nitrogen in subarctic peatlands. Glob. Change Biol..

[23] Liu X (2018). Nitrate is an important nitrogen source for Arctic tundra plants. Proc. Natl Acad. Sci. USA.

[24] Kou D (2020). Progressive nitrogen limitation across the Tibetan alpine permafrost region. Nat. Commun..

[25] Gao M (2019). Divergent changes in the elevational gradient of vegetation activities over the last 30 years. Nat. Commun..

[26] Salmon VG (2018). Adding depth to our understanding of nitrogen dynamics in permafrost soils. J. Geophys. Res. Biogeosci..

[27] Beaulieu JJ (2011). Nitrous oxide emission from denitrification in stream and river networks. Proc. Natl Acad. Sci. USA.

[28] Cooper RJ, Wexler SK, Adams CA, Hiscock KM (2017). Hydrogeological controls on regional-scale indirect nitrous oxide emission factors for rivers. Environ. Sci. Technol..

[29] Rosamond MS, Thuss SJ, Schiff SL (2012). Dependence of riverine nitrous oxide emissions on dissolved oxygen levels. Nat. Geosci..

[30] Venkiteswaran JJ, Rosamond MS, Schiff SL (2014). Nonlinear response of riverine N_2_O fluxes to oxygen and temperature. Environ. Sci. Technol..

[31] Zumft WG (1997). Cell biology and molecular basis of denitrification. Microbiol. Mol. Biol. Rev..

[32] Hallin S, Philippot L, Löffler FE, Sanford RA, Jones CM (2018). Genomics and ecology of novel N_2_O-reducing microorganisms. Trends Microbiol..

[33] Rees AP (2021). Biological nitrous oxide consumption in oxygenated waters of the high latitude Atlantic Ocean. Commun. Earth Environ..

[34] Alexander RB, Smith RA, Schwarz GE (2000). Effect of stream channel size on the delivery of nitrogen to the Gulf of Mexico. Nature.

[35] Gomez-Velez JD, Harvey JW (2014). A hydrogeomorphic river network model predicts where and why hyporheic exchange is important in large basins. Geophys. Res. Lett..

[36] Marzadri A, Dee MM, Tonina D, Bellin A, Tank JL (2017). Role of surface and subsurface processes in scaling N_2_O emissions along riverine networks. Proc. Natl Acad. Sci. USA.

[37] Xia X, Jia Z, Liu T, Zhang S, Zhang L (2017). Coupled nitrification-denitrification caused by suspended sediment (SPS) in rivers: Importance of SPS size and composition. Environ. Sci. Technol..

[38] Turner PA (2015). Indirect nitrous oxide emissions from streams within the US Corn Belt scale with stream order. Proc. Natl Acad. Sci. USA.

[39] Soued C, del Giorgio PA, Maranger R (2016). Nitrous oxide sinks and emissions in boreal aquatic networks in Québec. Nat. Geosci..

[40] Borges AV (2019). Variations in dissolved greenhouse gases (CO_2_, CH_4_, N_2_O) in the Congo River network overwhelmingly driven by fluvial-wetland connectivity. Biogeosciences.

[41] Allen GH, Pavelsky TM (2018). Global extent of rivers and streams. Science.

[42] Cavaliere E, Baulch HM (2018). Denitrification under lake ice. Biogeochemistry.

[43] Pepin N (2015). Elevation-dependent warming in mountain regions of the world. Nat. Clim. Change.

[44] Harms TK, Jones JB (2012). Thaw depth determines reaction and transport of inorganic nitrogen in valley bottom permafrost soils. Glob. Change Biol..

[45] Vonk JE (2015). Reviews and syntheses: effects of permafrost thaw on Arctic aquatic ecosystems. Biogeosciences.

[46] Tan E (2020). Warming stimulates sediment denitrification at the expense of anaerobic ammonium oxidation. Nat. Clim. Change.

[47] Yang X, Pavelsky TM, Allen GH (2020). The past and future of global river ice. Nature.

[48] Johnson KM, Hughes JE, Donaghay PL, Sieburth JM (1990). Bottle-calibration static head space method for the determination of methane dissolved in seawater. Anal. Chem..

[49] Lorke A (2015). Technical note: drifting versus anchored flux chambers for measuring greenhouse gas emissions from running waters. Biogeosciences.

[50] Campeau A, Lapierre J-F, Vachon D, del Giorgio PA (2014). Regional contribution of CO_2_ and CH_4_ fluxes from the fluvial network in a lowland boreal landscape of Québec. Glob. Biogeochem. Cycles.

[51] Wanninkhof R, Knox M (1996). Chemical enhancement of CO_2_ exchange in natural waters. Limnol. Oceanogr..

[52] Raymond PA (2012). Scaling the gas transfer velocity and hydraulic geometry in streams and small rivers. Limnol. Oceanogr. Fluids Environ..

[53] Alin, S. R. et al. Physical controls on carbon dioxide transfer velocity and flux in low-gradient river systems and implications for regional carbon budgets. *J. Geophys. Res. Biogeosci.*10.1029/2010JG001398 (2011).

[54] Wang Z (2016). Mapping the vegetation distribution of the permafrost zone on the Qinghai-Tibet Plateau. J. Mountain Sci..

[55] De’ath G, Fabricius KE (2000). Classification and regresstion trees: a powerful yet simple technique for ecological data analysis. Ecology.

[56] De’ath G (2002). Multivariate regression trees: a new technique for modeling species-environment relationships. Ecology.

[57] Obu, J., Westermann, S., Kääb, A. & Bartsch, A. *Permafrost Extent and Ground Temperature Map, 2000–2016, Northern Hemisphere Permafrost* (https://apgc.awi.de/dataset/pex). doi.pangaea.de/10.1594/PANGAEA.888600 (2018).

