# Supplementary material for: Unexpectedly minor nitrous oxide emissions from fluvial networks draining permafrost catchments of the East Qinghai-Tibet Plateau
Source: Nat Commun. 2022 Feb 17;13:950. doi: 10.1038/s41467-022-28651-8 (PMC8854393; doi:10.1038/s41467-022-28651-8)
Supplement: Supplementary file 1 — Supplementary Information [file 41467_2022_28651_MOESM1_ESM.pdf]

Supplementary Information for

Unexpectedly minor nitrous oxide emissions from fluvial networks draining permafrost catchments of the East Qinghai-Tibet Plateau

Liwei Zhang[1§,†], Sibo Zhang[2§], Xinghui Xia[1*], Tom J. Battin[3], Shaoda Liu[1], Qingrui Wang[1], Ran Liu[4], Zhifeng Yang[2], Jinren Ni[5] and Emily H. Stanley[6]

[1]Key Laboratory of Water and Sediment Sciences of Ministry of Education & State Key Laboratory of Water Environment Simulation, School of Environment, Beijing Normal University, Beijing, China

[2]Guangdong Provincial Key Laboratory of Water Quality Improvement and Ecological Restoration for Watersheds, Institute of Environmental and Ecological Engineering, Guangdong University of Technology, Guangzhou, China

[3]Stream Biofilm and Ecosystem Research Laboratory, School of Architecture, Civil and Environmental Engineering, École Polytechnique Fédérale de Lausanne, Lausanne, Switzerland

[4]Department of Mathematics, Beijing Jiaotong University, Beijing, China

[5]Key Laboratory of Water and Sediment Sciences of Ministry of Education, College of Environmental Sciences and Engineering, Peking University, Beijing, China

[6]Center for Limnology, University of Wisconsin-Madison, Madison, Wisconsin, USA

[§]Liwei Zhang and Sibo Zhang contributed equally to this work.

[†]Present address: Sino-French Institute for Earth System Science, College of Urban and Environmental Sciences, Peking University, Beijing, China.

*To whom correspondence may be addressed. Email: xiaxh@bnu.edu.cn.

This file includes:

Supplementary Methods

1. Dissolved N_2 concentrations.
2. Determination of benthic N_2O production rates.
3. Sample collection, DNA extraction, and real-time quantitative PCR.

Supplementary Discussion

1. Asynchronous seasonal patterns between concentrations and fluxes.
2. Anthropogenic N inputs on the QTP.
3. Patterns of gene abundances of (*nirS* + *nirK*) and *nosZ*.
4. Uncertainties.

Supplementary Figures

Fig 1. Names and locations of sampling stations (upper), and total vegetation cover (lower) in the four headwater watersheds of the EQTP, China.

Fig 2. Box plots of seasonal and regional patterns of N_2O concentrations (a) and fluxes (b) from EQTP rivers.

Fig 3. Photographs of intermediate runoff above the permafrost active layer in the Yangtze River Catchment.

Fig 4. DIN in relation to stream order across EQTP rivers.

Fig 5. Relationship between N_2O saturation and fluxes.

Fig 6. Relationships between dissolved oxygen saturation (%O_2) and gene abundances of (*nirS* + *nirK*) and *nosZ*.

Supplementary Tables

Table 1. Summary information on sampling sites.

Table 2. Characteristics of the headwater catchments on the EQTP.

Table 3. N_2O data for EQTP rivers and other lotic systems worldwide.

Table 4. Simple linear regression of N_2O concentration as functions of environmental variables.

Table 5. N_2O yields in EQTP rivers and existing reports for other lotic settings.

Table 6. Laboratory-measurement of benthic N_2O production rates by Yellow River sediments compared to measured field (*in situ*) fluxes and the estimated contribution of benthic fluxes to total N_2O emissions.

Table 7. Summary of *nir* and *nosZ* gene abundances for EQTP riverbed sediments and other lotic systems worldwide.

Table 8. The results of stepwise selection of predictive variables in multiple linear regression with N_2O flux.

Table 9. N_2O emissions in per unit stream/river surface area and basin area from EQTP waterways and other lotic settings worldwide.

Table 10. Primer pairs used in this study and corresponding amplification protocols.

Supplementary References

Supplementary Methods

1. Dissolved N_2 concentrations. Triplicate samples for dissolved N_2 were collected from surface water by completely filling 12 mL glass vials (Labco Exetainer®) at wrist depth below water surface at each site, then preserved by adding 100 μL saturated $ZnCl_2$ and stored at ambient temperature in the dark. Dissolved N_2 concentrations were analyzed with a membrane inlet mass spectrometer (MIMS; PrismaPlus®, Pfeiffer Vacuum)[1,2]. In brief, we measured ratios of N_2/Ar concentration using MIMS, then calculated Ar concentrations at *in situ* water temperature, pressure, and salinity[3]. Finally, dissolved N_2 concentrations were obtained by multiplying N_2/Ar with calculated Ar concentrations. The excess N_2 concentration (ΔN_2) was then calculated as: $\Delta N_2 = [N_2]_{measured} - [N_2]_{eq}$.

2. Determination of benthic N_2O production rates. In September 2018, nine sediment samples were collected in the Yellow River for sedimentary N_2O production rate determination. Briefly, approximately 10 g homogenized sediment samples were placed into 60 mL glass vials, and the remaining volume was filled with overlying water from the collection location. Before being transferred into glass vials, overlying water samples were adjusted to allow DO concentration in the laboratory to match field concentrations as closely as possible[4]. Vials were subsequently capped with silicone rubber septa and crimp-sealed with an aluminum closure then incubated at *in situ* temperature in the dark. The incubation vials were sacrificially sampled by injecting 300 μL of a saturated $ZnCl_2$ solution at 0, 4, 6, 10, and 18 h. At least four incubations were conducted for each specific sampling point, one of which was used to quantify changes in DO concentration, and the remaining replicates were used for N_2O analysis. N_2O concentrations in the glass vials were determined using the headspace equilibrium technique[5] on a GC-μECD. N_2O production rates were determined based on the linear increase of N_2O concentrations in the glass vials. Within the sampling period used for the N_2O production rate calculation, DO variation did not exceed 30% of its original concentration and final DO concentrations were consistently higher than 3.5 mg L^{-1}.

3. Sample collection, DNA extraction, and real-time quantitative PCR. A total of 26 riverbed sediment samples collected across the four rivers from 2016 to 2018 were prepared in triplicate. These samples were transported to the laboratory at -20 °C in a vehicle freezer prior to being stored at -80 °C in the laboratory. Genomic DNA was extracted from approximatively 0.5 g fresh homogenized sediment using the FastDNA® SPIN Kit for Soil (MP Biomedicals, USA) following the manufacturer's instructions; DNA extraction for each sample was performed in triplicate.

Real-time quantitative PCR (qPCR) was employed to estimate the abundances of dissimilatory nitrite reductase (*nirS* and *nirK*)[6] and clade I and II nitrous oxide reductase (*nosZ*) genes[7] in riverbed sediments. The *nirS* and *nirK* genes were amplified using currently available primer pair sets of cd3aF/R3cd and F1aCu/R3Cu, respectively[8]. Primer sets of nosZ2F/nosZ2R[9] and $nosZ_{II}$-F/$nosZ_{II}$-R[10], which encompass the known diversity of the *nosZ* gene, were used to quantify the $nosZ_I$ and $nosZ_{II}$ gene abundances. The 25 µL qPCR reaction mixtures consisted of 12.5 µL SYBR® Premix Ex Taq™ II (TaKaRa, Japan), 0.2 µL Bovine Serum Albumin (TaKaRa), 0.5 µL of each primer (5 µM), and 2 µL DNA template (~30–50 ng). All amplifications were performed in triplicate on a C1000™ Thermal Cycler (BioRad, CA, USA) according to the protocols described in Supplementary Table 10. The 10-fold serial dilutions of plasmid DNA containing cloned fragments of targeted genes were used to construct standard curves. Negative controls without DNA template were added to check potential DNA contamination. PCR amplification efficiencies for *nirS*, *nirK*, $nosZ_I$ and $nosZ_{II}$ genes all were higher than 85%, 90%, 86%, and 69%, respectively, with R^2 greater than 0.95 for each gene.

Supplementary Discussion

1. Asynchronous seasonal patterns between concentrations and fluxes.

The highest N_2O concentration occurred in spring, while the highest diffusive N_2O flux occurred in summer (Supplementary Fig. 2). This phenomenon is likely the result of temperature-driven differences in gas solubility. Cooler temperatures in the spring (ice-out season) led higher gas solubility and thus greater N_2O retention in the water column. In contrast, N_2O solubility was lower in warmer waters in summer. Once sediments are warmed and become saturated with N_2O, any additional N_2O delivered to and/or produced in channels readily escapes to the atmosphere. Lower N_2O concentration in summer may also be attributed to dilution due to seasonal maxima in precipitation (Supplementary Table 2), increased gas exchange, and reduced sediment-water contact.

In addition, a small number of our flux measurements were negative (i.e., N_2O entering the water from the atmosphere), yet all N_2O concentrations were supersaturated (Supplementary Fig. 5). The possible explanation may be that measured N_2O reflect transient concentrations, but N_2O consumption through complete denitrification may occur during 60-min floating chamber deployments. This hypothesis is consistent with results of low N_2O yield, small ratio of *nir*/*nos*, and laboratory benthic production rates.

2. Anthropogenic N inputs on the QTP.

Tibetan nomadic herdsmen have been active on the QTP for thousands of years, so livestock grazing is the most important anthropogenic influence on the plateau's biogeochemical cycles[11]. But human population densities are low at higher altitudes (Supplementary Table 2), because of the harsh environmental stressors that are distinct from lowlands[12]. Likewise, domestic livestock (yaks and sheep) are bred in lower altitude basins, whereas wild animals (Tibetan antelopes, Tibetan gazelles, kiangs, and wild yaks) are distributed at higher altitudes. There is a strong negative correlation between distribution of livestock and wild animals, illustrating that their habitats rarely overlap due to the competition for forage resources[13]. Based on human population density in each catchment

(Supplementary Table 2), we deduce that manure N in the Yangtze and Upper Yellow Catchments was mainly derived from wild animals (natural sources); manure N in the Nu and Lancang Catchments came from both wild animals and domestic livestock (mixed sources); manure N in the Lower Yellow Catchment originated from livestock and belonged to anthropogenic sources.

The DIN pool in EQTP rivers is dominated by NO_3^- (represent an average of 86% of DIN), and anthropogenic NO_3^- inputs to aquatic systems on the QTP are derived from human sewage, livestock manure, and synthetic fertilizers. Among these sources, livestock manure N is currently the largest input to EQTP waterways. However, manure largely represents internal source of N in the alpine grassland systems, as it originates from plant N uptake from soils. Stable isotopes of NO_3^- ($\delta^{15}N$, $\Delta^{17}O$ and $\delta^{18}O$) combined with the above deduction indicate that sites underlain by continuous permafrost in the Yangtze Catchment receive substantial natural NO_3^- inputs from permafrost soils (47.1 ± 1.2%), wild animal manure (33.4 ± 3.3%), and atmospheric precipitation (18.5 ± 1.3%), but little anthropogenic NO_3^- input from sewage (1.0 ± 0.8%) and none from livestock manure or fertilizers[14]. By comparison, anthropogenic NO_3^- inputs from livestock manure (34.9 ± 10.0%), sewage (5.6 ± 2.3%), and fertilizers (14.9 ± 5.4%) became dominant in the Lancang (site LTJ) and Lower Yellow Rivers in non-continuous permafrost zone, and the contribution of natural NO_3^- inputs [permafrost soils (37.7 ± 3.9%) and atmospheric precipitation (6.9 ± 1.6%)] were reduced accordingly[14,15]. Above all, both natural and anthropogenic N can be taken up by plants, thus a part of terrestrial N can be sequestered before entering rivers.

3. Patterns of gene abundances of (*nirS* + *nirK*) and *nosZ*.

The relationship in Supplementary Fig. 6 was driven by a decline in gene abundances of *nir* genes when $\%O_2 > 110\%$, while *nos* gene abundances were not apparently responsive to the degree of $\%O_2$. When $\%O_2 \geq 100\%$, *nos* of some denitrifiers is still functional at the transcriptional and metabolic levels, albeit at low rates, and may even have a higher tolerance to oxygen than *nir*[16,17].

4. Uncertainties.

Upscaling N_2O emissions from headwater streams is a challenging task because the contribution of 1st- and 2nd- order streams to total efflux incorporates uncertainties in both gas fluxes and surface area. To reduce the uncertainty associated with the estimate of the total surface area of 1st- and 2nd- order streams, we surveyed river width in the headwater fluvial networks from 3rd- to 7th- order across the EQTP in combination with extracting 50 width measurement from each order (1–7) from Google Earth Map. We assumed that stream lengths derived from GIS were constant and that error from this step was not propagated through to the upscaling. Our approach yielded a mean 1st stream channel width of 1.78 m, which is similar with the stream width reported by Downing and colleagues[18], but wider than those reported more recently by Allen and colleagues[19]. A possible explanation for this difference may be that channel width to depth ratios at most of our sites are extremely high (> 60; see Supplementary Table 1), indicating that channels on the EQTP tend to adjust to increases in discharge by becoming wider rather than deeper.

Although our sampled streams and rivers were constrained to 3rd–7th order sites on the EQTP, N_2O fluxes were measured in four catchments with distinct alpine landscape and different amounts of permafrost areas, capturing at least part of the spatiotemporal heterogeneity of the fluvial networks. In addition, a Monte Carlo simulation based on our data was used to provide upper and lower limits on the total flux estimate of 1st–7th order streams and rivers to bound the uncertainty. Despite current uncertainties, our range of the total value given for the region seems reasonable.

Supplementary Figures

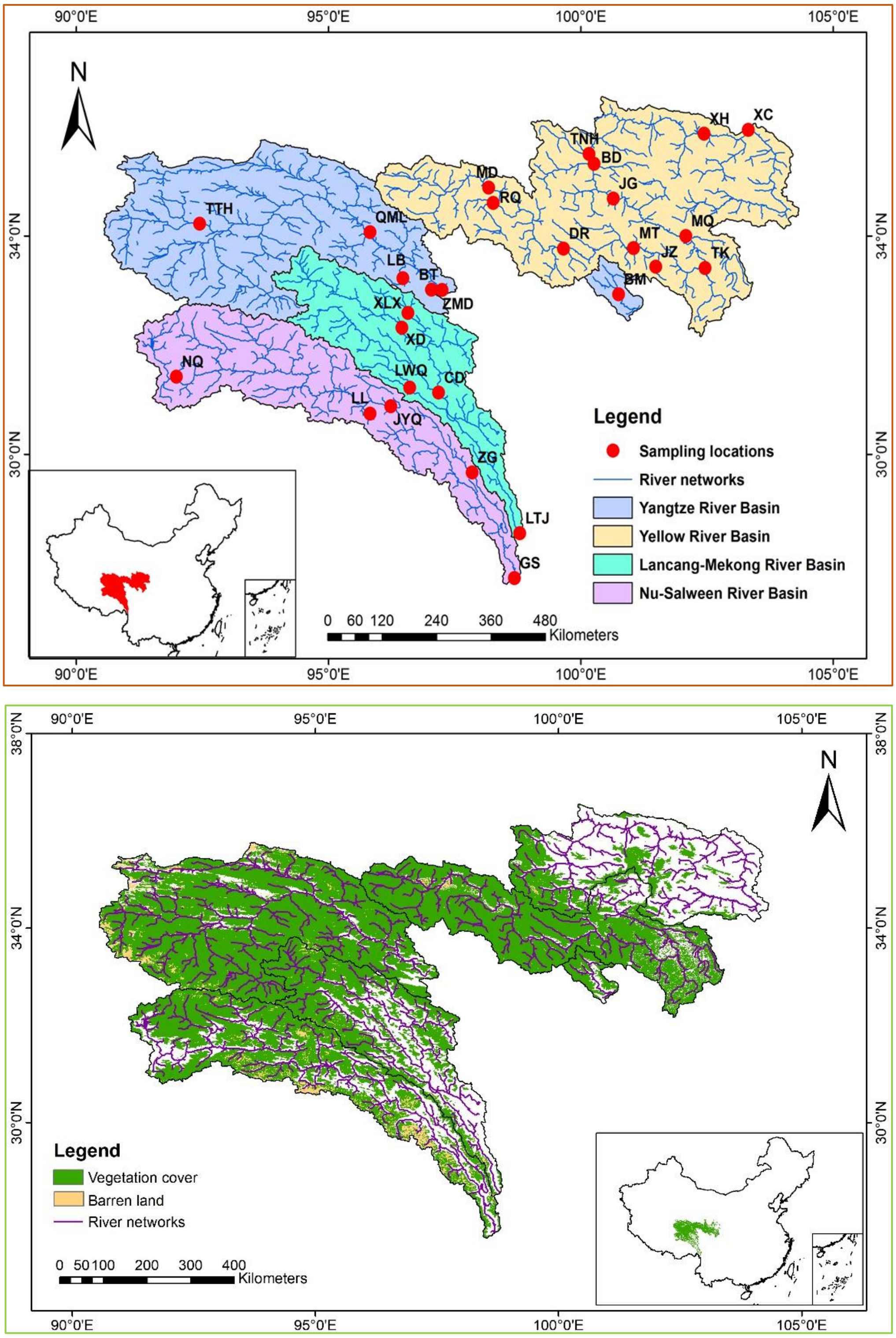

Supplementary Figure 1 | Map of the four headwater basins on the East Qinghai-Tibet Plateau (EQTP), China. Names and locations of sampling stations (**upper**), and total vegetation cover (**lower**) in the four headwater basins of the EQTP.

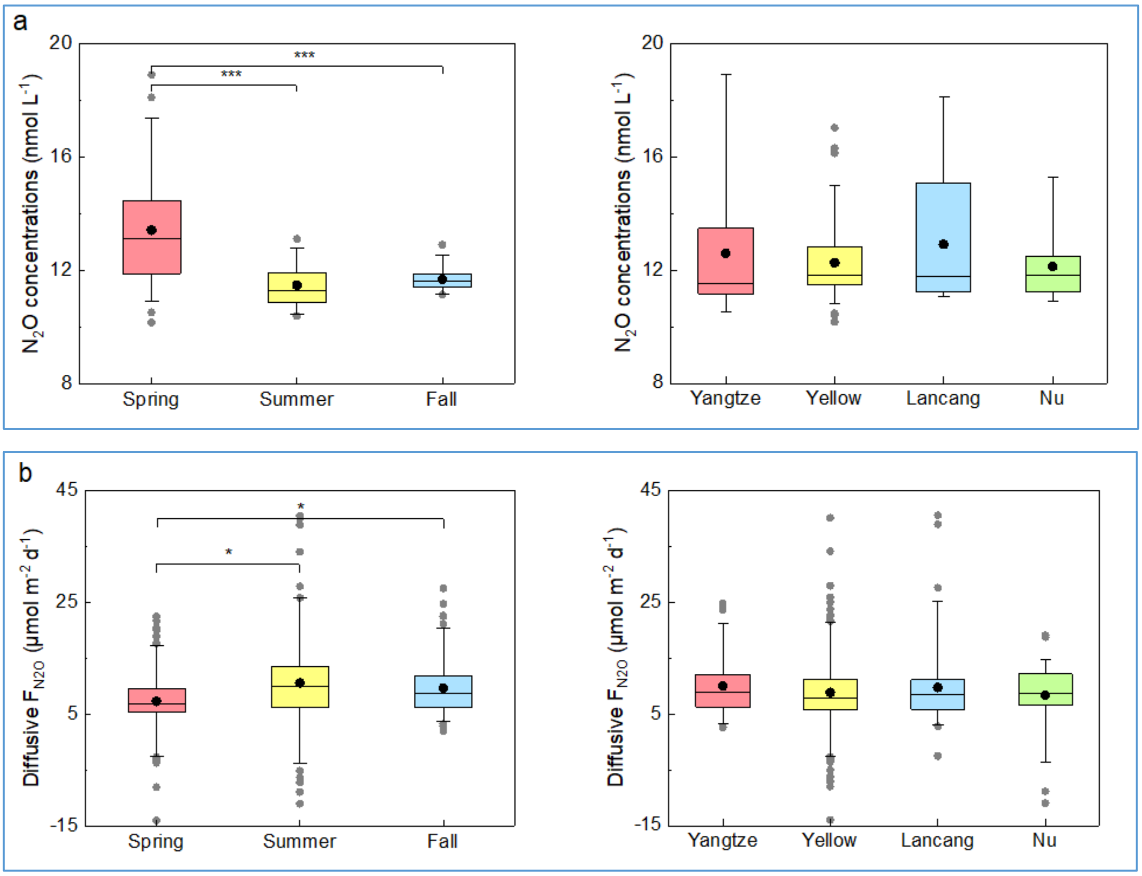

Supplementary Figure 2 | Box plots of seasonal and regional patterns of N_2O concentrations (a) and fluxes (b) from EQTP rivers. Boxes represent the 25th and 75th percentiles, and error bars show the 95th percentiles; black circles and horizontal lines indicate the arithmetic means and medians, respectively. Grey circles are outliers. (one-way ANOVA with Tukey post hoc test, $^{*}P < 0.05$; $^{**}P < 0.01$; $^{***}P < 0.001$).

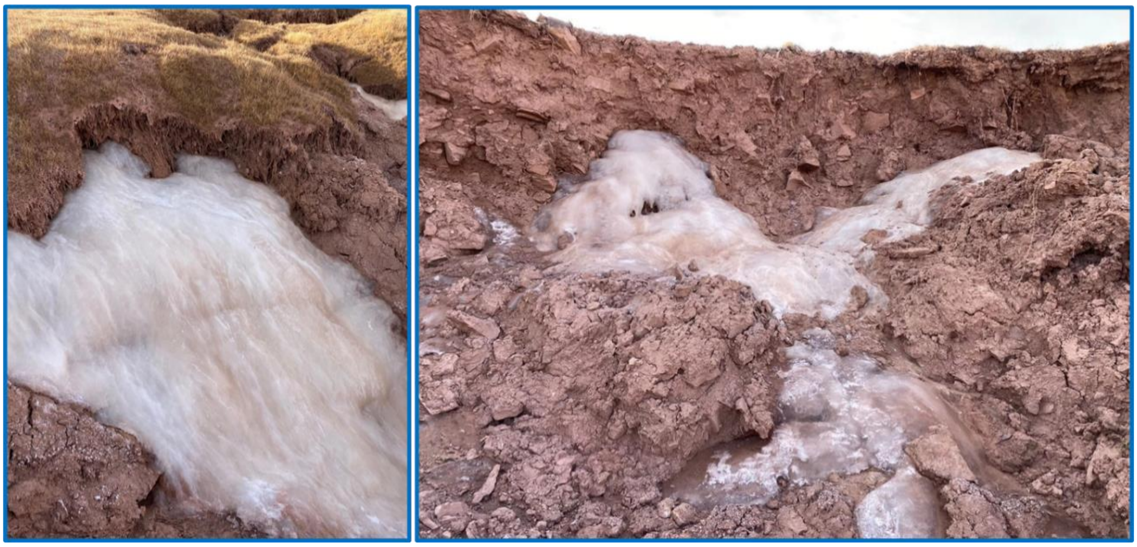

Supplementary Figure 3 | Photographs of intermediate runoff above the permafrost active layer in the Yangtze River Catchment. The photos were taken in late October 2020.

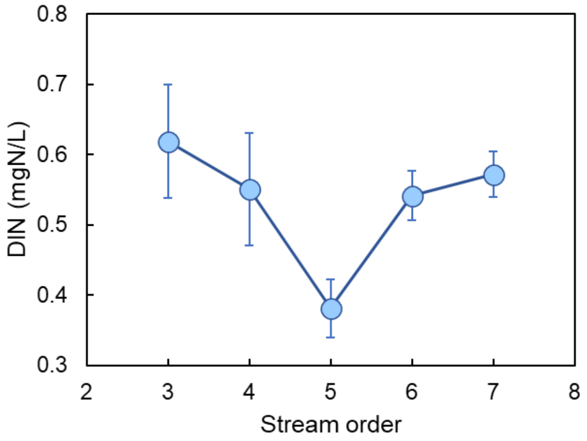

Supplementary Figure 4 | DIN in relation to stream order across EQTP rivers. Data points are the means and error bars represent ± 1 SE.

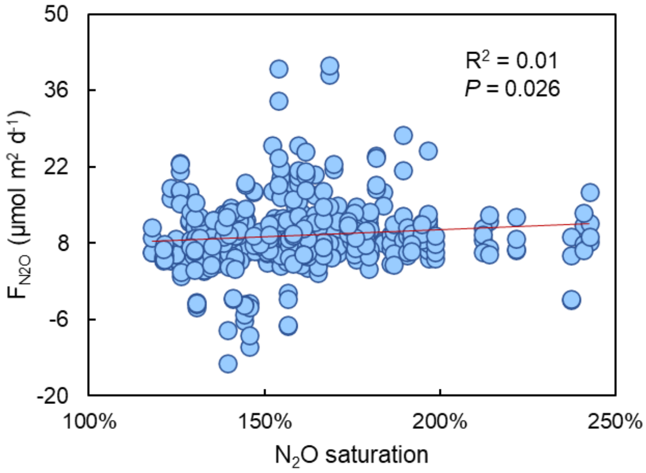

Supplementary Figure 5 | Relationship between N_2O saturation and fluxes. The red line represents the fit of a linear regression through the observed data.

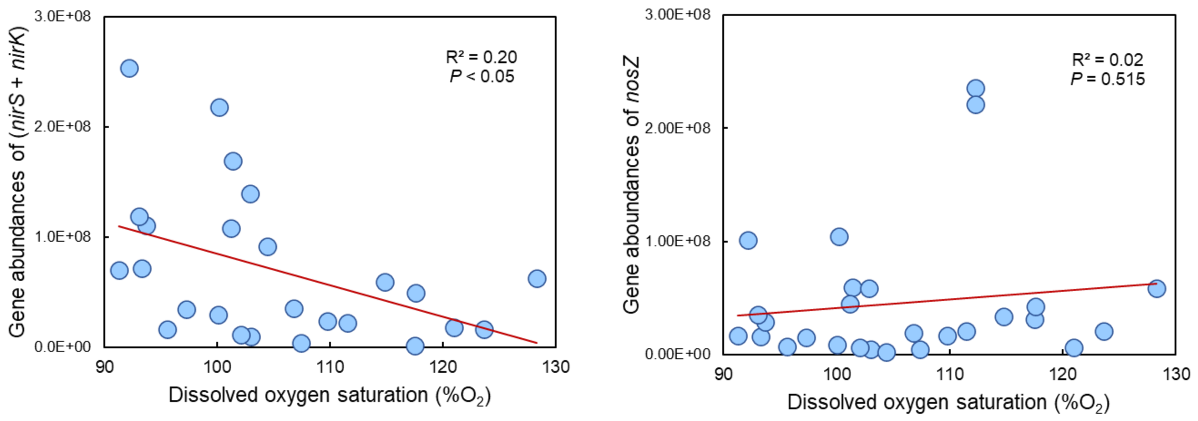

Supplementary Figure 6 | Relationships between dissolved oxygen saturation (%O_2) and gene abundances of (*nirS* + *nirK*) and *nosZ*. The red lines represent the fit of linear regressions through the observed data.

Supplementary Tables

Supplementary Table 1. Summary information on sampling sites (see Supplementary Fig. 1 for site locations).

Headwater Basins		Sampling Sites	Longitude (E)	Latitude (N)	Stream/river Reaches	Strahler Stream Order	Elevation (m)	Water depth (m)	River width (m)	Flow velocity (m/s)	Discharge (m^3/s)	Sediment type	SS concentration (mg/L)
Yangtze River	Mainstem	TTH	92°26'37"	34°13'15"	Tuotuo River	6	4550	0.34	75.5	0.72	18.5	Muck	99
		QML	95°49'17"	34°04'01"	Upper Tongtian River	7	4080	2.07 ± 1.06	174 ± 44.5	1.67 ± 0.7	711.9 ± 790.5	Muck	198
		ZMD	97°14'50"	33°00'32"	Lower Tongtian River	7	3540	2.59 ± 1.31	157 ± 15.7	1.89 ± 0.73	847.3 ± 906.3	Gravel/cobble	208
	Tributaries	LB	96°28'23"	33°13'26"	Longbao Creek	3	4210	0.64	15.4	1.2	9.28	Gravel/cobble	7
		BT	97°02'28"	33°00'56"	Batang Stream	4	3674	0.63 ± 0.18	37.0 ± 9.12	1.41 ± 0.4	34.2 ± 9.68	Gravel/cobble	29
		BM	100°44'45"	32°55'43"	Markog River	5	3555	0.6 ± 0.12	61.2 ± 21.2	1.08 ± 0.04	57 ± 38.2	Muck/sand	79
Yellow River	Mainstem	MD	98°10'16"	34°53'08"	Uppermost Yellow River	5	4274	0.65 ± 0.44	43.1 ± 29.8	0.65 ± 0.13	24.4 ± 55	Muck	27 ± 30.4
		DR	99°39'24"	33°46'02"	Upper Yellow River	6	4008	0.97 ± 0.26	93.5 ± 28	1.23 ± 0.2	146.2 ± 122.8	Muck	90.4 ± 92.5
		MT	101°02'39"	33°46'30"	Upper Yellow River	6	3715	1.91 ± 0.46	108 ± 23.5	1.42 ± 0.47	283.3 ± 265.8	Fine sand	30.8 ± 15.6
		MQ	102°04'47"	33°59'46"	Upper Yellow River	6	3469	2.4 ± 0.88	257 ± 11.5	0.69 ± 0.19	553 ± 457.4	Muck	144.5 ± 73.6
		JG	100°38'42"	34°41'02"	Lower Yellow River	7	3126	2.2 ± 1.01	153 ± 15.1	1.95 ± 0.39	694.8 ± 573.6	Coarse sand	328.4 ± 277.4
		BD	100°15'49"	35°19'17"	Lower Yellow River	7	2760	1.83 ± 0.23	140 ± 12.5	1.73 ± 0.68	509 ± 127.7	Fine sand	628 ± 673.1
		TNH	100°09'51"	35°29'60"	Lower Yellow River	7	2711	2.67 ± 1.1	146 ± 2.79	2.1 ± 0.1	886.8 ± 2.79	Coarse sand	354.5 ± 337.3
		XH	102°26'41"	35°52'13"	Lower Yellow River	7	1880	2.67 ± 0.24	118 ± 7	1.32 ± 0.38	463 ± 200.2	Gravel/cobble	23.7 ± 17.4
		XC	103°19'02"	35°56'22"	Lower Yellow River	7	1650	4.5 ± 1.06	146 ± 1.53	1.14 ± 0.65	934 ± 464.5	/	40 ± 14.1
	Tributaries	RQ	98°15'52"	34°36'05"	Re Qu	5	4255	0.66 ± 0.39	81.3 ± 3.99	0.73 ± 0.2	35 ± 44.9	Fine sand	19.7 ± 23.1
		JZ	101°28'59"	33°25'58"	Sharkog Stream	3	3653	0.55 ± 0.19	39.5 ± 3.48	1.12 ± 0.3	21.8 ± 21.6	Muck	15.8 ± 2.3
		TK	102°27'42"	33°24'38"	White River	5	3520	0.56 ± 0.26	225 ± 28.6	0.63 ± 0.13	62.3 ± 36.6	Muck	56 ± 11.9
Lancang River	Mainstem	XD	96°27'07"	32°18'53"	Zha Qu	6	3690	1.45 ± 0.4	104 ± 3.39	1.47 ± 0.11	248 ± 112.9	Coarse sand	194.3 ± 36.1
		CD	97°10'44"	31°07'38"	Uppermost Lancang River	7	3190	3.61 ± 0.7	89.9 ± 10.7	2.28 ± 0.4	846.3 ± 442	Gravel/cobble	198
		LTJ	98°47'24"	28°33'08"	Upper Lancang River	7	2079	8.67 ± 1.69	70.5 ± 9.79	2.38 ± 1.74	1141.7 ± 544.1	/	241
	Tributaries	XLX	96°34'17"	32°35'20"	Zi Qu	4	3798	0.96 ± 0.09	46.8 ± 6.88	1.34 ± 0.13	65 ± 27	Gravel/cobble	38.3 ± 21.4
		LWQ	96°36'24"	31°13'17"	Purple Stream	5	3803	1.09 ± 0.2	66.3 ± 3.67	1.64 ± 0.17	124.7 ± 58.4	Coarse sand	89 ± 17
Nu River	Mainstem	NQ	91°58'54"	31°25'20"	Na Qu	5	4601	0.83	80.5	1.38	79.6	Muck	15.6
		JYQ	96°14'01"	30°52'38"	Upper Nu River	7	3198	6.33 ± 0.85	89.7 ± 3.18	2.35±0.21	1390 ± 340.4	Coarse sand	153
		GS	98°41'	27°44'	Mid Nu River	7	1712	9.52 ± 0.83	114 ± 4.95	1.97	2167.5 ± 512.7	Fine sand	174
	Tributaries	LL	95°49'24"	30°44'30"	Dromarangtso Creek	4	3639	0.63 ± 0.15	17.9 ± 7.02	1.7 8± 0.23	17.9 ± 7.02	Gravel/cobble	39 ± 34.8
		ZG	97°50'49"	29°40'10"	Yu Qu	5	3775	0.76 ± 0.26	71.6 ± 6.26	1.32 ± 0.68	71.6 ± 6.26	Fine sand	104.3 ± 0.72

Supplementary Table 2. Characteristics of the headwater catchments on the EQTP.

	Yangtze	Upper Yellow	Nu	Lancang	Lower Yellow
Catchment area (km^2)	216,108	193,016	143,255	106,996	76,995
Mean catchment altitude (m)	3,935	3,842	3,385	3,312	2,425
Permafrost coverage (km^2)	205,735	140,130	87,959	56,264	16,400
Permafrost fraction	95.2%	72.6%	61.4%	52.6%	21.3%
Vegetation coverage (km^2)*	184,988	154,220	87,099	60,667	18,941
Vegetation fraction	85.6%	79.9%	60.8%	56.7%	24.6%
Barren land (km^2)*	9,295	4,878	11,904	3,072	966
Barren land fraction	4.3%	2.5%	8.3%	2.9%	1.3%
Human population (× 10^4)†	28.8	20.1	36.2	39.3	65.0
Population density (km^{-2})	1.3	1.3	2.5	3.7	8.4
Annual temperature (°C)§	1.4	1.2	7.1	6.4	5.1
Annual precipitation (mm)§	1,855.8	2,768.3	2,102.3	1,619.3	1,541.7
Spring	609.3	858.9	745.5	491.7	436.4
Summer	718.3	1,086.0	845.2	709.4	792.7
Fall	528.2	823.4	511.6	418.2	312.6
**Means (± standard deviation) of physicochemical property measured in streams and rivers**					
DOC (mgC/L)	7.18 ± 3.26	5.34 ± 2.08	4.42 ± 2.61	4.52 ± 1.94	5.24 ± 2.35
DIN (mgN/L)	0.62 ± 0.22	0.44 ± 0.23	0.47 ± 0.23	0.59 ± 0.19	0.71 ± 0.51
TP (mg/L)	0.25 ± 0.36	0.12 ± 0.14	0.23 ± 0.23	0.14 ± 0.12	0.25 ± 0.43
pH	8.22 ± 0.16	8.34 ± 0.20	8.37 ± 0.17	8.22 ± 0.12	8.40 ± 0.16
DO (mg/L)	6.87 ± 0.89	6.87 ± 0.68	7.13 ± 0.89	6.97 ± 0.67	7.59 ± 0.61
Conductivity (µS/cm)	1003.8 ± 784.5	277.2 ± 202.5	486.5 ± 194.2	227.2 ± 194.7	398.8 ± 98.2
ORP (mV)	150.8 ± 26.0	151.7 ± 56.2	139.1 ± 31.6	140.4 ± 21.2	154.0 ± 98.8
Water temperature (°C)	12.3 ± 3.5	12.1 ± 3.4	14.6 ± 2.6	13.6 ± 3.1	13.4 ± 3.1

Sampling Time

	2016			2017		2018	
	Spring	Summer	Fall	Spring	Summer	Spring	Fall
Yangtze				√	√	√	√
Yellow	√	√	√	√	√	√	√
Lancang					√	√	√
Nu					√	√	√

Note that the Yellow River was divided into Upper and Lower Yellow Catchments at JG site based on the permafrost fraction (Supplementary Fig. 1 and Table 1).

*Vegetation coverage and barren land was calculated from ref [20].

†Human population was obtained from National Bureau of Statistics (www.stats.gov.cn/).

[§]Average annual temperature and precipitation was obtained from National Meteorological Information Center (http://data.cma.cn/).

Supplementary Table 3. N_2O data for EQTP rivers and other lotic systems worldwide.

Latitude	Regions	Lotic Systems	N_2O			$EF_{5\text{-}r} = \frac{N_2O\text{-}N}{NO_3^-\text{-}N}$ (mass ratio)	Ref.
			Concentrations (nM)	Fluxes [μmol/(m^2·d)]			
				Diffusion	Ebullition		
< 24°	Sub-Saharan Africa	Sub-Saharan African rivers	9.2	13.0	/	/	21
		Congo River	7.9	22.0	/	0.28%	22
	Kenya	Mara River	18.2	13.7	/	0.05%	23
	Malaysia	Malaysian rivers	11.7	21.3	/	0.10%	24,25
	Ecuador	Cuenca River	/	30.0		/	26
24-54°	South Asia	Adyar River	26.5	21.0	/	0.26%	27
		Ganges River	31.2	13.7		0.03%	28
		Sênggê Tsangpo-Indus River	0.21 μatm	4.6	/	/	29
	Southeast Asia	Mekong River	30.7	13.6	/	0.14%	28
	China	Jiulong River	59.1	13.5	/	0.03%	30
		Wu River	33.3	15.3	/	0.16%	31
		Yongan River	16.9	20.1	/	0.04%	32
		Chongqing river network	113.8	261.6	/	0.47%	33
		Shanghai river network	/	68.2	/	/	34
		Beijing river network	42.5	83.4	/	0.10%	35
		Yarlung Tsangpo	13.4	5.7–13.2	/	0.17%	36
		EQTP rivers	12.4 (0.34 μatm)	9.4	0.74	0.17%	This study
		Yangtze River (lowland)	15.9	12.1	/	0.04%	37
		Yellow River (lowland)	22.4	42.6	/	0.03%	38
		Xilin River	/	24.3	/	/	39
	UK	Upper Thurne River	82.3	129.8	/	0.27%	40
		Wensum, Eden & Avon River	51.7	50.0	/	0.024%	41
	France	Seine River	36.8	69.9	/	0.02%	42,43
	Belgium	Meuse River	42.9	/	/	0.37%	44
	Canada	Grand River	/	-35–4,200	/	/	45
		Ontario streams	/	-3.2–776	< 0.004	/	46,47
	USA	San Joaquin River	32.5	8.1–318.9	/	0.28%	48
		Agricultural streams in Illinois	71.4	102.9	/	/	49
		Hudson River	0.58 μatm	5.5	/	/	50
		Kalamazoo River	28.9	30.2	/	0.20%	51
		Connecticut River	15.4	28.9	/	0.22%	52
		Lamprey River	0.8 μatm	46.8	/	/	53

		Upper Mississippi River	/	0.72	/	/	54
		Streams within the Corn Belt	/	0.03–49.7	/	/	55
		Agricultural headwater streams	/	41.8	/		
		Urban headwater streams	/	49.2	/	0.75%	56
		Pristine headwater streams	/	4.2	/		
	Australia	New South Waters	/	4.0	/	/	57
	New Zealand	LII river	46.9	96.7	7.9 µL/L	0.03%	58,59
		Ashburton River	12.3	14.6–27	/	0.06%	60
> 54°	Sweden	Swedish low-order streams	50.0	141.5	/	0.63%	61,62
	Canada	Québec streams & rivers	5.9	9.4	/	0.86%	63
	USA	Water tracks within the Upper Kuparuk River	/	-10.3	/	/	64
	Global streams and rivers		37.5	94.3	/	0.22%	65

Note that grey shaded are mountain streams and rivers.

Supplementary Table 4. Simple linear regression of N_2O concentration as functions of environmental variables. R^2 values are shown for regressions with *P* values. Direction of correlations is indicated as '+' for positive and '–' for negative. Significant relationships are shaded.

Environmental variables	R^2	*P* value	Direction of the correlation
Air pressure	0.02	> 0.05	+
Water temperature	0.02	> 0.05	–
pH	0.004	> 0.05	+
DO	0.004	> 0.05	–
$\%O_2$	0.2	< 0.001	–
DOC	0.03	0.049	–
NH_4^+	0.1	< 0.001	+
NO_3^-	0.23	< 0.001	+
Log TP	0.07	0.004	+

Supplementary Table 5. N_2O yields in EQTP rivers and existing reports for other lotic settings.

Lotic settings	N_2O yields (%)		Ref.
	Mean	Range	
Lower Yangtze River	0.82	0.51–1.12	37
Yellow River (lowland)	1.13	0.06–6.24	38
Beijing river networks	1.6	0.01–23.1	35
US headwater streams	0.9	0.04–5.6	56
Choptank & Nanticoke River	2.60	0.48–6.11	66
Tippecanoe River	0.94	0.78–1.1	67
Kalamazoo River	12.0	0.9–53.8	68
Seine River	1.5	≤ 7.0	69
UK estuaries	0.7	0.52–0.77	70
EQTP rivers	0.23	0.003–0.87	This study

Supplementary Table 6. Laboratory-measurement of benthic N_2O production rates by the Yellow River sediments compared to measured field (*in situ*) fluxes and the estimated contribution of benthic fluxes to total N_2O emissions.

	Sites	Lab benthic F_{N2O} (μmol m^{-2} d^{-1})	*in situ* F_{N2O} (μmol m^{-2} d^{-1})	Contribution (%)
Permafrost-rich sites	MD	-1.45	13.44	-10.8
	RQ	-0.62	14.06	-4.4
	DR	-0.76	8.77	-8.7
	MT*	22.14	10.83	204.4
	JZ	-1.37	9.14	-15.0
	MQ*	17.06	18.56	91.9
	TK	0.04	7.08	0.6
Permafrost-poor sites	JG	-0.69	8.85	-7.8
	TNH	-1.24	9.90	-12.5

*The ratios of (*nirS* + *nirK*)/*nosZ* for MT and MQ were 2.6 times higher than those for other sites, suggesting the N_2O yields were higher at the two sites.

Supplementary Table 7. Summary of *nir* and *nosZ* gene abundances for EQTP riverbed sediments and other lotic systems worldwide.

Lotic systems	Functional gene abundances (copies/g dw)				Ratios		Ref.
	nirS	*nirK*	$nosZ_{I}$	$nosZ_{II}$	$(nirS + nirK)/nosZ_{I}$	$(nirS + nirK)/nosZ$	
Pearl River	166×10^8	7.12×10^8	8.05×10^8	/	21.5	21.5	[71]
Nanfei River	3.6×10^8	0.8×10^8	1.3×10^8	/	3.38	3.38	[72]
Tama River*	3.31×10^5	3.80×10^5	3.10×10^5	0.19×10^5	2.29	2.16	[73]
Olentangy River	14.3×10^8	2.1×10^8	0.3×10^8	/	54.7	54.7	[74]
Deba River	9.17×10^{11}	6.95×10^9	2.93×10^5	/	3.24×10^6	3.24×10^6	[75]
Rhône River	2.4×10^7	0.2×10^7	0.1×10^7	0.7×10^7	26.0	3.25	[76]
Garonne River	5.2×10^9	6.3×10^9	0.72×10^9	/	16.0	16.0	[77]
EQTP rivers	6.32×10^7	2.60×10^7	4.44×10^7	0.11×10^7	2.01	1.96	This study

Note that some studies failed to quantify $nosZ_{II}$, so we also compared our ratio of $(nirS + nirK)/nosZ_{I}$ with this subset of references. Our ratio is still the smallest, even lower than those for Tama River.

*Functional gene abundances for the Tama River (copies/L) were measured in the overlying water instead of sediments, so both functional genes abundances and ratios of $(nirS + nirK)/nosZ$ were much lower than those of other studies. Despite this sampling difference, the ratios still exceed that observed in EQTP rivers.

Supplementary Table 8. The results of stepwise selection of predictive variables in multiple linear regression with N_2O flux.

Environmental variables	Unstandardized Coefficients		Standardized Coefficients	*t*	Sig.
	Beta	Std. Error	Beta		
%O_2	-0.150	0.028	-0.259	-5.352	< 0.001
pH	3.078	0.721	0.202	4.270	< 0.001
Water temperature	0.235	0.072	0.155	3.271	0.001
TP	-1.491	0.560	-0.132	-2.665	0.008
NO_3^-	2.569	1.113	0.112	2.308	0.022
Constant	-6.369	6.938		-0.918	0.359

Supplementary Table 9. N_2O emissions in per unit stream/river surface area and basin area from EQTP waterways and other lotic settings worldwide.

Streams & rivers	Stream/river surface area (km^2)	Basin Area ($\times 10^4$ km^2)	N_2O emissions (GgN_2O-N/yr)	Per unit river surface area [tN_2O-N/($km^2\cdot yr$)]	Per unit basin area [kgN_2O-N/($km^2\cdot yr$)]	Percentage of N_2O in GHG emissions	Ref.
Congo River*	23,209	370.5	5.16	0.22	1.39	0.2%	[22]
Malaysian rivers	790	6.06	0.35	0.44	5.78	4.6%	[25,78]
Adyar River	6.9	0.05	1.53×10^{-3}	0.22	2.89	7.2%	[27,79]
Yongan River	/	0.25	6.56×10^{-3}	/	2.65	/	[32]
Shanghai river network	570	/	0.29	0.51	/	2.8%	[34,80]
Beijing river network	216	/	0.14	0.65	/	13.9%	[35,81]
Seine River	283	7.17	0.24	0.85	3.35	4.9%	[42,43]
Rabbit River	3.6	0.07	1.09×10^{-3}	0.30	1.56	/	[51]
Grand River	/	0.68	0.01	/	1.37	/	[45]
Swedish low-order streams	697	/	1.78	2.55	/	7.5%	[62,82]
Boreal rivers in Québec*	1,445	20.4	0.11	0.08	0.55	1.2%	[63,83]
EQTP 3rd–7th rivers*	2,603	73.6	0.21	0.08	0.28	1.0%	This study and ref.[84]
EQTP 1st–7th waterways*	3,049		0.28	0.09	0.37	0.4%	
QTP 1st–7th waterways*	5,141	116.7	0.43–0.46	0.09	0.37	/	

*The Congo River, Québec rivers and QTP rivers are pristine (limited anthropogenic influence) rivers; the remaining rivers are human-impacted systems.

Supplementary Table 10. **Primer pairs used in this study and corresponding amplification protocols.**

Specificity	Primer	Sequence (5'–3')	Thermal conditions	Ref.
nirS	cd3af	GTSAACGTSAAGGARACSGG	(95 °C, 3 min) × 1<br>(95 °C, 30 s; 58 °C, 40 s; 72 °C, 40 s) × 40	8
	R3cd	GASTTCGGRTGSGTCTTGA		
nirK	F1aCu	ATCATGGTSCTGCCGCG	(95 °C, 3 min) × 1<br>(95 °C, 30 s; 60 °C, 30 s; 72 °C, 40 s) × 40	
	R3Cu	GCCTCGATCAGRTTGTGGTT		
nosZ$_{I}$	nosZ2F	CGCRACGGCAASAAGGTSMSSGT	(95 °C, 3 min) × 1<br>(95 °C, 30 s; 60 °C, 30 s; 72 °C, 30 s) × 40	9
	nosZ2R	CAKRTGCAKSGCRTGGCAGAA		
nosZ$_{II}$	nosZ$_{II}$-F	CTIGGICCIYTKCAYAC	(95 °C, 3 min) × 1<br>(95 °C, 30 s; 54 °C, 45 s; 72 °C, 45 s) × 40	10
	nosZ$_{II}$-R	GCIGARCARAAITCBGTRC		

Supplementary References

1 Kana, T. M. *et al.* Membrane inlet mass spectrometer for rapid high-precision determination of N_2, O_2, and Ar in environmental water samples. *Analytical Chemistry* **66**, 4166-4170, doi:10.1021/ac00095a009 (1994).

2 Laursen, A. E. & Seitzinger, S. P. Measurement of denitrification in rivers: an integrated, whole reach approach. *Hydrobiologia* **485**, 67-81, doi:10.1023/A:1021398431995 (2002).

3 Weiss, R. F. The solubility of nitrogen, oxygen and argon in water and seawater. *Deep Sea Research and Oceanographic Abstracts* **17**, 721-735, doi:10.1016/0011-7471(70)90037-9 (1970).

4 Zhang, S. *et al.* Both microbial abundance and community composition mattered for N2 production rates of the overlying water in one high-elevation river. *Environmental Research* **189**, 109933, doi:10.1016/j.envres.2020.109933 (2020).

5 Johnson, K. M., Hughes, J. E., Donaghay, P. L. & Sieburth, J. M. Bottle-calibration static head space method for the determination of methane dissolved in seawater. *Analytical Chemistry* **62**, 2408-2412, doi:10.1021/ac00220a030 (1990).

6 Zumft, W. G. Cell biology and molecular basis of denitrification. *Microbiology and Molecular Biology Reviews* **61**, 533-616 (1997).

7 Hallin, S., Philippot, L., Löffler, F. E., Sanford, R. A. & Jones, C. M. Genomics and ecology of novel N_2O-reducing microorganisms. *Trends in Microbiology* **26**, 43-55, doi:10.1016/j.tim.2017.07.003 (2018).

8 Throbäck, I. N., Enwall, K., Jarvis, Å. & Hallin, S. Reassessing PCR primers targeting *nirS*, *nirK* and *nosZ* genes for community surveys of denitrifying bacteria with DGGE. *FEMS Microbiology Ecology* **49**, 401-417, doi:10.1016/j.femsec.2004.04.011 (2004).

9 Henry, S., Bru, D., Stres, B., Hallet, S. & Philippot, L. Quantitative detection of the *nosZ* gene, encoding nitrous oxide reductase, and comparison of the abundances of 16S rRNA, *narG*, *nirK*, and *nosZ* genes in soils. *Applied and Environmental Microbiology* **72**, 5181-5189, doi:10.1128/aem.00231-06 (2006).

10 Jones, C. M., Graf, D. R. H., Bru, D., Philippot, L. & Hallin, S. The unaccounted yet abundant nitrous oxide-reducing microbial community: a potential nitrous oxide sink. *The ISME Journal* **7**, 417-426, doi:10.1038/ismej.2012.125 (2013).

11 Chen, H. *et al.* The impacts of climate change and human activities on biogeochemical cycles on the Qinghai-Tibetan Plateau. *Global Change Biology* **19**, 2940-2955, doi:10.1111/gcb.12277 (2013).

12 Tremblay, J. C. & Ainslie, P. N. Global and country-level estimates of human population at high altitude. *Proceedings of the National Academy of Sciences* **118**, e2102463118, doi:10.1073/pnas.2102463118 (2021).

13 Li, X., Gao, E., Li, B. & Zhan, X. Estimating abundance of Tibetan wild ass, Tibetan gazelle and Tibetan antelope using species distribution model and distance sampling (in Chinese). *SCIENTIA SINICA Vitae* **49**, 151, doi:10.1360/N052018-00171 (2019).

14 Xia, X. *et al.* Triple oxygen isotopic evidence for atmospheric nitrate and its application in source identification for river systems in the Qinghai-Tibetan Plateau. *Science of The Total Environment* **688**, 270-280, doi:10.1016/j.scitotenv.2019.06.204 (2019).

15 Guo, X. *et al.* Using stable nitrogen and oxygen isotopes to identify nitrate sources in the Lancang River, upper Mekong. *Journal of Environmental Management* **274**, 111197, doi:10.1016/j.jenvman.2020.111197 (2020).

16 Körner, H. & Zumft, W. G. Expression of denitrification enzymes in response to the dissolved oxygen

level and respiratory substrate in continuous culture of *Pseudomonas stutzeri*. *Applied and Environmental Microbiology* **55**, 1670-1676 (1989).
17 Qu, Z., Bakken, L. R., Molstad, L., Frostegård, Å. & Bergaust, L. L. Transcriptional and metabolic regulation of denitrification in *Paracoccus denitrificans* allows low but significant activity of nitrous oxide reductase under oxic conditions. *Environmental Microbiology* **18**, 2951-2963, doi:10.1111/1462-2920.13128 (2016).
18 Downing, J. A. *et al.* Global abundance and size distribution of streams and rivers. *Inland Waters* **2**, 229-236, doi:10.5268/IW-2.4.502 (2012).
19 Allen, G. H. *et al.* Similarity of stream width distributions across headwater systems. *Nature Communications* **9**, 610, doi:10.1038/s41467-018-02991-w (2018).
20 Wang, Z. *et al.* Mapping the vegetation distribution of the permafrost zone on the Qinghai-Tibet Plateau. *Journal of Mountain Science* **13**, 1035-1046, doi:10.1007/s11629-015-3485-y (2016).
21 Borges, A. V. *et al.* Globally significant greenhouse-gas emissions from African inland waters. *Nature Geoscience* **8**, 637-642, doi:10.1038/ngeo2486 (2015).
22 Borges, A. V. *et al.* Variations in dissolved greenhouse gases (CO_2, CH_4, N_2O) in the Congo River network overwhelmingly driven by fluvial-wetland connectivity. *Biogeosciences* **16**, 3801-3834, doi:10.5194/bg-16-3801-2019 (2019).
23 Mwanake, R. M. *et al.* Land use, not stream order, controls N_2O concentration and flux in the Upper Mara River Basin, Kenya. *Journal of Geophysical Research: Biogeosciences* **124**, 3491-3506, doi:10.1029/2019JG005063 (2019).
24 Müller, D. *et al.* Nitrous oxide and methane in two tropical estuaries in a peat-dominated region of northwestern Borneo. *Biogeosciences* **13**, 2415-2428, doi:10.5194/bg-13-2415-2016 (2016).
25 Bange, H. W. *et al.* Nitrous oxide (N_2O) and methane (CH_4) in rivers and estuaries of northwestern Borneo. *Biogeosciences* **16**, 4321-4335, doi:10.5194/bg-16-4321-2019 (2019).
26 Ho, L. *et al.* Effects of land use and water quality on greenhouse gas emissions from an urban river system. *Biogeosciences Discuss.* **2020**, 1-22, doi:10.5194/bg-2020-311 (2020).
27 Nirmal Rajkumar, A., Barnes, J., Ramesh, R., Purvaja, R. & Upstill-Goddard, R. C. Methane and nitrous oxide fluxes in the polluted Adyar River and estuary, SE India. *Marine Pollution Bulletin* **56**, 2043-2051, doi:10.1016/j.marpolbul.2008.08.005 (2008).
28 Begum, M. S. *et al.* Localized pollution impacts on greenhouse gas dynamics in three anthropogenically modified Asian river systems. *Journal of Geophysical Research: Biogeosciences* **126**, e2020JG006124, doi:10.1029/2020JG006124 (2021).
29 Qu, B. *et al.* Greenhouse gases emissions in rivers of the Tibetan Plateau. *Scientific Reports* **7**, 16573, doi:10.1038/s41598-017-16552-6 (2017).
30 Chen, N., Wu, J., Zhou, X., Chen, Z. & Lu, T. Riverine N_2O production, emissions and export from a region dominated by agriculture in Southeast Asia (Jiulong River). *Agriculture, Ecosystems & Environment* **208**, 37-47, doi:10.1016/j.agee.2015.04.024 (2015).
31 Liang, X. *et al.* Control of the hydraulic load on nitrous oxide emissions from cascade reservoirs. *Environmental Science & Technology* **53**, 11745-11754, doi:10.1021/acs.est.9b03438 (2019).
32 Hu, M. *et al.* Modeling riverine N_2O sources, fates, and emission factors in a typical river network of Eastern China. *Environmental Science & Technology* **55**, 13356-13365, doi:10.1021/acs.est.1c01301 (2021).
33 He, Y. *et al.* Effect of watershed urbanization on N_2O emissions from the Chongqing metropolitan river network, China. *Atmospheric Environment* **171**, 70-81, doi:10.1016/j.atmosenv.2017.09.043

(2017).
34 Yu, Z. *et al.* Nitrous oxide emissions in the Shanghai river network: Implications for the effects of urban sewage and IPCC methodology. *Global Change Biology* **19**, 2999-3010, doi:10.1111/gcb.12290 (2013).
35 Wang, G. *et al.* Distinctive patterns and controls of nitrous oxide concentrations and fluxes from urban inland waters. *Environmental Science & Technology* **55**, 8422-8431, doi:10.1021/acs.est.1c00647 (2021).
36 Ye, R. *et al.* Concentrations and emissions of dissolved CH_4 and N_2O in the Yarlung Tsangpo River. *Chinese Journal of Ecology* **38**, 791-798 (2019).
37 Yan, W., Yang, L., Wang, F., Wang, J. & Ma, P. Riverine N_2O concentrations, exports to estuary and emissions to atmosphere from the Changjiang River in response to increasing nitrogen loads. *Global Biogeochemical Cycles* **26**, doi:10.1029/2010gb003984 (2012).
38 Xia, X. *et al.* Nitrogen loss from a turbid river network based on N_2 and N_2O fluxes: Importance of suspended sediment. *Science of The Total Environment* **757**, 143918, doi:10.1016/j.scitotenv.2020.143918 (2021).
39 Hao, X. *et al.* Greenhouse gas emissions from the water-air interface of a grassland river: a case study of the Xilin River. *Scientific Reports* **11**, 2659, doi:10.1038/s41598-021-81658-x (2021).
40 Outram, F. N. & Hiscock, K. M. Indirect nitrous oxide emissions from surface water bodies in a lowland arable catchment: A significant contribution to agricultural greenhouse gas budgets? *Environmental Science & Technology* **46**, 8156-8163, doi:10.1021/es3012244 (2012).
41 Cooper, R. J., Wexler, S. K., Adams, C. A. & Hiscock, K. M. Hydrogeological controls on regional-scale indirect nitrous oxide emission factors for rivers. *Environmental Science & Technology* **51**, 10440-10448, doi:10.1021/acs.est.7b02135 (2017).
42 Garnier, J. *et al.* Nitrous oxide (N_2O) in the Seine river and basin: Observations and budgets. *Agriculture, Ecosystems & Environment* **133**, 223-233, doi:10.1016/j.agee.2009.04.024 (2009).
43 Marescaux, A., Thieu, V. & Garnier, J. Carbon dioxide, methane and nitrous oxide emissions from the human-impacted Seine watershed in France. *Science of The Total Environment* **643**, 247-259, doi:10.1016/j.scitotenv.2018.06.151 (2018).
44 Borges, A. V. *et al.* Effects of agricultural land use on fluvial carbon dioxide, methane and nitrous oxide concentrations in a large European river, the Meuse (Belgium). *Science of The Total Environment* **610-611**, 342-355, doi:10.1016/j.scitotenv.2017.08.047 (2018).
45 Rosamond, M. S., Thuss, S. J. & Schiff, S. L. Dependence of riverine nitrous oxide emissions on dissolved oxygen levels. *Nature Geoscience* **5**, 715-718, doi:10.1038/ngeo1556 (2012).
46 Baulch, H. M., Schiff, S. L., Maranger, R. & Dillon, P. J. Nitrogen enrichment and the emission of nitrous oxide from streams. *Global Biogeochemical Cycles* **25**, doi:10.1029/2011gb004047 (2011).
47 Baulch, H. M., Dillon, P. J., Maranger, R. & Schiff, S. L. Diffusive and ebullitive transport of methane and nitrous oxide from streams: Are bubble-mediated fluxes important? *Journal of Geophysical Research: Biogeosciences* **116**, n/a-n/a, doi:10.1029/2011JG001656 (2011).
48 Hinshaw, S. E. & Dahlgren, R. A. Dissolved nitrous oxide concentrations and fluxes from the eutrophic San Joaquin River, California. *Environmental Science & Technology* **47**, 1313-1322, doi:10.1021/es301373h (2013).
49 Davis, M. P. & David, M. B. Nitrous oxide fluxes from agricultural streams in east-central Illinois. *Water, Air, & Soil Pollution* **229**, 354, doi:10.1007/s11270-018-4007-7 (2018).
50 Cole, J. J. & Caraco, N. F. Emissions of nitrous oxide (N_2O) from a tidal, freshwater river, the Hudson

River, New York. *Environmental Science & Technology* **35**, 991-996, doi:10.1021/es0015848 (2001).
51 Beaulieu, J. J., Arango, C. P., Hamilton, S. K. & Tank, J. L. The production and emission of nitrous oxide from headwater streams in the Midwestern United States. *Global Change Biology* **14**, 878-894, doi:10.1111/j.1365-2486.2007.01485.x (2008).
52 Aho, K. S. *et al.* An intense precipitation event causes a temperate forested drainage network to shift from N_2O source to sink. *Limnology and Oceanography* **n/a**, doi:10.1002/lno.12006.
53 Schade, J. D., Bailio, J. & McDowell, W. H. Greenhouse gas flux from headwater streams in New Hampshire, USA: Patterns and drivers. *Limnology and Oceanography* **61**, S165-S174, doi:10.1002/lno.10337 (2016).
54 Turner, P. A. *et al.* Regional-scale controls on dissolved nitrous oxide in the Upper Mississippi River. *Geophysical Research Letters* **43**, 4400-4407, doi:10.1002/2016gl068710 (2016).
55 Turner, P. A. *et al.* Indirect nitrous oxide emissions from streams within the US Corn Belt scale with stream order. *Proceedings of the National Academy of Sciences* **112**, 9839-9843, doi:10.1073/pnas.1503598112 (2015).
56 Beaulieu, J. J. *et al.* Nitrous oxide emission from denitrification in stream and river networks. *Proceedings of the National Academy of Sciences* **108**, 214-219, doi:10.1073/pnas.1011464108 (2011).
57 Andrews, L. F. *et al.* Hydrological, geochemical and land use drivers of greenhouse gas dynamics in eleven sub-tropical streams. *Aquatic Sciences* **83**, 40, doi:10.1007/s00027-021-00791-x (2021).
58 Clough, T. J., Bertram, J. E., Sherlock, R. R., Leonard, R. L. & Nowicki, B. L. Comparison of measured and $EF_{5\text{-}r}$-derived N_2O fluxes from a spring-fed river. *Global Change Biology* **12**, 352-363, doi:10.1111/j.1365-2486.2005.01089.x (2006).
59 Clough, T. J., Buckthought, L. E., Kelliher, F. M. & Sherlock, R. R. Diurnal fluctuations of dissolved nitrous oxide (N_2O) concentrations and estimates of N_2O emissions from a spring-fed river: implications for IPCC methodology. *Global Change Biology* **13**, 1016-1027, doi:10.1111/j.1365-2486.2007.01337.x (2007).
60 Clough, T. J., Buckthought, L. E., Casciotti, K. L., Kelliher, F. M. & Jones, P. K. Nitrous oxide dynamics in a braided river system, New Zealand. *Journal of Environmental Quality* **40**, 1532-1541, doi:10.2134/jeq2010.0527 (2011).
61 Audet, J., Wallin, M. B., Kyllmar, K., Andersson, S. & Bishop, K. Nitrous oxide emissions from streams in a Swedish agricultural catchment. *Agriculture, Ecosystems & Environment* **236**, 295-303, doi:10.1016/j.agee.2016.12.012 (2017).
62 Audet, J. *et al.* Forest streams are important sources for nitrous oxide emissions. *Global Change Biology* **26**, 629-641, doi:10.1111/gcb.14812 (2020).
63 Soued, C., del Giorgio, P. A. & Maranger, R. Nitrous oxide sinks and emissions in boreal aquatic networks in Québec. *Nature Geoscience* **9**, 116-120, doi:10.1038/ngeo2611 (2016).
64 Harms, T. K., Rocher-Ros, G. & Godsey, S. E. Emission of greenhouse gases from water tracks draining Arctic hillslopes. *Journal of Geophysical Research: Biogeosciences* **125**, e2020JG005889, doi:10.1029/2020JG005889 (2020).
65 Hu, M., Chen, D. & Dahlgren, R. A. Modeling nitrous oxide emission from rivers: A global assessment. *Global Change Biology* **22**, 3566-3582, doi:10.1111/gcb.13351 (2016).
66 Gardner, J. R., Fisher, T. R., Jordan, T. E. & Knee, K. L. Balancing watershed nitrogen budgets: accounting for biogenic gases in streams. *Biogeochemistry* **127**, 231-253, doi:10.1007/s10533-015-0177-1 (2016).

67 Dee, M. M. & Tank, J. L. Inundation time mediates denitrification end products and carbon limitation in constructed floodplains of an agricultural stream. *Biogeochemistry*, doi:10.1007/s10533-020-00670-x (2020).

68 Beaulieu, J. J., Arango, C. P. & Tank, J. L. The effects of season and agriculture on nitrous oxide production in headwater streams. *Journal of Environmental Quality* **38**, 637-646, doi:10.2134/jeq2008.0003 (2009).

69 Billen, G. *et al.* Modeling indirect N_2O emissions along the N cascade from cropland soils to rivers. *Biogeochemistry* **148**, 207-221, doi:10.1007/s10533-020-00654-x (2020).

70 Dong, L. F., Nedwell, D. B. & Stott, A. Sources of nitrogen used for denitrification and nitrous oxide formation in sediments of the hypernutrified Colne, the nutrified Humber, and the oligotrophic Conwy estuaries, United Kingdom. *Limnology and Oceanography* **51**, 545-557, doi:10.4319/lo.2006.51.1_part_2.0545 (2006).

71 Huang, S., Chen, C., Yang, X., Wu, Q. & Zhang, R. Distribution of typical denitrifying functional genes and diversity of the *nirS*-encoding bacterial community related to environmental characteristics of river sediments. *Biogeosciences* **8**, 3041-3051, doi:10.5194/bg-8-3041-2011 (2011).

72 Zhang, M. *et al.* Response of chemical properties, microbial community structure and functional genes abundance to seasonal variations and human disturbance in Nanfei River sediments. *Ecotoxicology and Environmental Safety* **183**, 109601, doi:10.1016/j.ecoenv.2019.109601 (2019).

73 Thuan, N. C. *et al.* N_2O production by denitrification in an urban river: evidence from isotopes, functional genes, and dissolved organic matter. *Limnology* **19**, 115-126, doi:10.1007/s10201-017-0524-0 (2018).

74 Ligi, T. *et al.* Effects of soil chemical characteristics and water regime on denitrification genes (*nirS*, *nirK*, and *nosZ*) abundances in a created riverine wetland complex. *Ecological Engineering* **72**, 47-55, doi:10.1016/j.ecoleng.2013.07.015 (2014).

75 Martínez-Santos, M. *et al.* Treated and untreated wastewater effluents alter river sediment bacterial communities involved in nitrogen and sulphur cycling. *Science of The Total Environment* **633**, 1051-1061, doi:10.1016/j.scitotenv.2018.03.229 (2018).

76 Mahamoud Ahmed, A., Lyautey, E., Bonnineau, C., Dabrin, A. & Pesce, S. Environmental concentrations of copper, alone or in mixture with arsenic, can impact river sediment microbial community structure and functions. *Frontiers in Microbiology* **9**, doi:10.3389/fmicb.2018.01852 (2018).

77 Lyautey, E. *et al.* Abundance, activity and structure of denitrifier communities in phototrophic river biofilms (River Garonne, France). *Hydrobiologia* **716**, 177-187, doi:10.1007/s10750-013-1561-2 (2013).

78 Wit, F. *et al.* The impact of disturbed peatlands on river outgassing in Southeast Asia. *Nature Communications* **6**, 10155, doi:10.1038/ncomms10155 (2015).

79 Panneer Selvam, B., Natchimuthu, S., Arunachalam, L. & Bastviken, D. Methane and carbon dioxide emissions from inland waters in India – implications for large scale greenhouse gas balances. *Global Change Biology* **20**, 3397-3407, doi:10.1111/gcb.12575 (2014).

80 Yu, Z. *et al.* Carbon dioxide and methane dynamics in a human-dominated lowland coastal river network (Shanghai, China). *Journal of Geophysical Research: Biogeosciences* **122**, 1738-1758, doi:10.1002/2017jg003798 (2017).

81 Wang, G. *et al.* Intense methane ebullition from urban inland waters and its significant contribution to greenhouse gas emissions. *Water Research* **189**, 116654, doi:10.1016/j.watres.2020.116654 (2021).

82 Wallin, M. B. *et al.* Carbon dioxide and methane emissions of Swedish low-order streams—A national estimate and lessons learnt from more than a decade of observations. *Limnology and Oceanography Letters* **3**, 156-167, doi:10.1002/lol2.10061 (2018).

83 Hutchins, R. H. S., Casas-Ruiz, J. P., Prairie, Y. T. & del Giorgio, P. A. Magnitude and drivers of integrated fluvial network greenhouse gas emissions across the boreal landscape in Québec. *Water Research* **173**, 115556, doi:10.1016/j.watres.2020.115556 (2020).

84 Zhang, L. *et al.* Significant methane ebullition from alpine permafrost rivers on the East Qinghai-Tibet Plateau. *Nature Geoscience* **13**, 349-354, doi:10.1038/s41561-020-0571-8 (2020).
