# Supplementary material for: Unexpectedly minor nitrous oxide emissions from fluvial networks draining permafrost catchments of the East Qinghai-Tibet Plateau
Source: Nat Commun. 2022 Feb 17;13:950. doi: 10.1038/s41467-022-28651-8 (PMC8854393; doi:10.1038/s41467-022-28651-8)
Supplement: Supplementary file 3 — Reporting Summary [file 41467_2022_28651_MOESM3_ESM.pdf]

nature portfolio

Corresponding author(s): Xinghui Xia

Last updated by author(s): Jan 21, 2022

Reporting Summary

Nature Portfolio wishes to improve the reproducibility of the work that we publish. This form provides structure for consistency and transparency in reporting. For further information on Nature Portfolio policies, see our Editorial Policies and the Editorial Policy Checklist.

Statistics

For all statistical analyses, confirm that the following items are present in the figure legend, table legend, main text, or Methods section.

n/a	Confirmed	
☐	☒	The exact sample size (*n*) for each experimental group/condition, given as a discrete number and unit of measurement
☐	☒	A statement on whether measurements were taken from distinct samples or whether the same sample was measured repeatedly
☐	☒	The statistical test(s) used AND whether they are one- or two-sided<br>*Only common tests should be described solely by name; describe more complex techniques in the Methods section.*
☒	☐	A description of all covariates tested
☐	☒	A description of any assumptions or corrections, such as tests of normality and adjustment for multiple comparisons
☐	☒	A full description of the statistical parameters including central tendency (e.g. means) or other basic estimates (e.g. regression coefficient) AND variation (e.g. standard deviation) or associated estimates of uncertainty (e.g. confidence intervals)
☐	☒	For null hypothesis testing, the test statistic (e.g. F, t, r) with confidence intervals, effect sizes, degrees of freedom and P value noted<br>*Give P values as exact values whenever suitable.*
☒	☐	For Bayesian analysis, information on the choice of priors and Markov chain Monte Carlo settings
☒	☐	For hierarchical and complex designs, identification of the appropriate level for tests and full reporting of outcomes
☒	☐	Estimates of effect sizes (e.g. Cohen's d, Pearson's r), indicating how they were calculated

Our web collection on statistics for biologists contains articles on many of the points above.

Software and code

Policy information about availability of computer code

Data collection	No software was used in this study for data collection.
Data analysis	The Monte Carlo model and regression tree analysis code used in this study were performed by MATLAB R2018b. Pearson correlations and multiple linear stepwise regression analysis were conducted under the Statistical Product and Service Solutions 25.0 software (SPSS) at a significance level of P < 0.05. Details were reported in "Upscaling technique" and "Statistical analyses" in the Methods section.

For manuscripts utilizing custom algorithms or software that are central to the research but not yet described in published literature, software must be made available to editors and reviewers. We strongly encourage code deposition in a community repository (e.g. GitHub). See the Nature Portfolio guidelines for submitting code & software for further information.

Data

Policy information about availability of data

All manuscripts must include a data availability statement. This statement should provide the following information, where applicable:

- Accession codes, unique identifiers, or web links for publicly available datasets
- A description of any restrictions on data availability
- For clinical datasets or third party data, please ensure that the statement adheres to our policy

The data used in this study are available at the Environmental Data Initiative (https://portal.edirepository.org/nis/mapbrowse?packageid=edi.695.1), doi:10.6073/pasta/ba9340800403c450e7d942d450237dc4.

Field-specific reporting

Please select the one below that is the best fit for your research. If you are not sure, read the appropriate sections before making your selection.

☐ Life sciences ☐ Behavioural & social sciences ☒ Ecological, evolutionary & environmental sciences

For a reference copy of the document with all sections, see nature.com/documents/nr-reporting-summary-flat.pdf

Ecological, evolutionary & environmental sciences study design

All studies must disclose on these points even when the disclosure is negative.

Study description	We directly measured N2O concentrations and fluxes during different seasons between 2016 and 2018 in four watersheds on the East Qinghai-Tibet Plateau, and estimated that fluvial N2O emissions from permafrost landscapes on the entire Qinghai-Tibet Plateau was small.
Research sample	All samples were obtained from a broad range of permafrost-affected streams and rivers (that ranged from Strahler order 3 to 7) in the headwater basins of Yellow, Yangtze, Lancang and Nu Rivers on the Qinghai-Tibet Plateau. These samples may have broader implications for aquatic N2O dynamics in streams and rivers at high latitudes and altitudes.
Sampling strategy	We collected gas, water and sediment samples in four headwater catchments that vary in altitude from 1,650 to 4,600 m and cover an area of ca. 73.6 × 104 km2 on the East Qinghai-Tibet Plateau from May 2016 to Sep 2018.
Data collection	Data collection procedure was described in "N2O concentrations and fluxes" in the Methods section. Liwei Zhang recorded the data on paper and/or laboratory apparatus.
Timing and spatial scale	The sampling occurred between May 2016 and Sep 2018, and covered different seasons (spring, summer and autumn) as far as possible. The exact dates for each site are available at the Environmental Data Initiative (https://portal.edirepository.org/nis/mapbrowse?packageid=edi.695.1), doi:10.6073/pasta/ba9340800403c450e7d942d450237dc4.
Data exclusions	Grey points in Fig. 4a denote samples affected by reservoirs, and were excluded from the correlation analysis.
Reproducibility	Samples for dissolved N2O concentrations and fluxes were collected from different locations in each site/transect to capture the spatial heterogeneity. Duplicate samples were used for physicochemical analyses, and triplicate samples were used for microbial analyses. All attempts to repeat the experiment were successful.
Randomization	We grouped the sampling sites into four categories that represent different permafrost zones: continuous, discontinuous, sporadic, and isolated. We then merged discontinuous, sporadic, and isolated permafrost zones together under non-continuous permafrost group in response to different NDVI trends (see Fig. 2b).<br>All data of N2O concentrations were divided into three subgroups according to regression tree analysis as displayed in Fig. 3b.
Blinding	We did not predict the results in advance, all data were analyzed based on actual conditions.

Did the study involve field work? ☒ Yes ☐ No

Field work, collection and transport

Field conditions	Please see "Site description" in the Method section, and Fig. 1; Supplementary Fig. 1, Table 1 and 2 for details.
Location	Please see "Site description" in the Method section, and Fig. 1; Supplementary Fig. 1, Table 1 and 2 for details.
Access & import/export	The surveys were done by car, with sampling from boats, bridges, or from the bank with the help of hydrologic stations.
Disturbance	The floating chambers used in this study were of the same size and shape and streamlined with a flexible plastic foil collar to minimize the effects of chamber-induced turbulence when measuring fluxes and were covered with aluminum foil to reflect the sunlight and minimize internal heating.

Reporting for specific materials, systems and methods

We require information from authors about some types of materials, experimental systems and methods used in many studies. Here, indicate whether each material, system or method listed is relevant to your study. If you are not sure if a list item applies to your research, read the appropriate section before selecting a response.

Materials & experimental systems

n/a	Involved in the study
☒	☐ Antibodies
☒	☐ Eukaryotic cell lines
☒	☐ Palaeontology and archaeology
☒	☐ Animals and other organisms
☒	☐ Human research participants
☒	☐ Clinical data
☒	☐ Dual use research of concern

Methods

n/a	Involved in the study
☒	☐ ChIP-seq
☒	☐ Flow cytometry
☒	☐ MRI-based neuroimaging
